# EZH2‐mediated inhibition of KLF14 expression promotes HSCs activation and liver fibrosis by downregulating PPARγ

**DOI:** 10.1111/cpr.13072

**Published:** 2021-05-24

**Authors:** Zhipeng Du, Mei Liu, Zhihui Wang, Zhuoying Lin, Yangyang Feng, Dean Tian, Limin Xia

**Affiliations:** ^1^ Hubei Key Laboratory of Hepato‐Pancreato‐Biliary Diseases Department of Gastroenterology Institute of Liver and Gastrointestinal Diseases Tongji Hospital of Tongji Medical College Huazhong University of Science and Technology Wuhan China

**Keywords:** enhancer of zeste homolog 2, hepatic stellate cell, krüppel‐like factor 14, liver fibrosis, peroxisome proliferator‐activated receptor γ

## Abstract

**Objectives:**

Induction of deactivation and apoptosis of hepatic stellate cells (HSCs) are principal therapeutic strategies for liver fibrosis. Krüppel‐like factor 14 (KLF14) regulates various biological processes, however, roles, mechanisms and implications of KLF14 in liver fibrosis are unknown.

**Materials and Methods:**

KLF14 expression was detected in human, rat and mouse fibrotic models, and its effects on HSCs were assessed. Chromatin immunoprecipitation assays were utilized to investigate the binding of KLF14 to peroxisome proliferator‐activated receptor γ (PPARγ) promoter, and the binding of enhancer of zeste homolog 2 (EZH2) to KLF14 promoter. In vivo, KLF14‐overexpressing adenovirus was injected via tail vein to thioacetamide (TAA)‐treated rats to investigate the role of KLF14 in liver fibrosis progression. EZH2 inhibitor EPZ‐6438 was utilized to treat TAA‐induced rat liver fibrosis.

**Results:**

KLF14 expression was remarkably decreased in human, rat and mouse fibrotic liver tissues. Overexpression of KLF14 increased LD accumulation, inhibited HSCs activation, proliferation, migration and induced G2/M arrest and apoptosis. Mechanistically, KLF14 transactivated PPARγ promoter activity. Inhibition of PPARγ blocked the suppressive role of KLF14 overexpression in HSCs. Downregulation of KLF14 in activated HSCs was mediated by EZH2‐regulated histone H3 lysine 27 trimethylation. Adenovirus‐mediated KLF14 overexpression ameliorated TAA‐induced rat liver fibrosis in PPARγ‐dependent manner. Furthermore, EPZ‐6438 dramatically alleviated TAA‐induced rat liver fibrosis. Importantly, KLF14 expression was decreased in human with liver fibrosis, which was significantly correlated with EZH2 upregulation and PPARγ downregulation.

**Conclusions:**

KLF14 exerts a critical anti‐fibrotic role in liver fibrosis, and targeting the EZH2/KLF14/PPARγ axis might be a novel therapeutic strategy for liver fibrosis.

## Graphical abstract

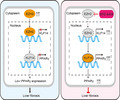

## 1 INTRODUCTION

Various chronic liver diseases cause liver fibrosis, and advanced liver fibrosis leads to liver cirrhosis, which results in approximately 1.16 million deaths per year worldwide. Liver fibrosis also generates a permissive niche for the development of hepatocellular carcinoma.^1^, ^2^ Therefore, it is urgent to uncover the underlying mechanism of liver fibrogenesis and develop effective targets for better anti‐fibrotic therapies. Liver fibrosis is featured by excessive accumulation of extracellular matrix (ECM), and the central event for this process is hepatic stellate cells (HSCs) activation.^3^, ^4^ Under physiological condition, HSCs are quiescent and the most distinctive feature is abundant vitamin A stored on cytoplasmic lipid droplets (LD),^5^ and this phenotype is mainly maintained by several adipogenic transcription factors, such as peroxisome proliferator‐activated receptor γ (PPARγ), CCAAT/enhancer‐binding proteins (C/EBPs), liver X receptor α (LXRα) and sterol regulatory element‐binding protein 1c (SREBP‐1c).^6^, ^7^ Upon activation, the quiescent HSCs transdifferentiate to myofibroblasts, which are fibrogenic, proliferative, contractile and chemotactic, accompanied by rapid loss of LD.^8^ Recent studies reported that recovery of the LD content in activated HSCs could convert the activated HSCs to the quiescent phenotype, and induction of HSCs deactivation and apoptosis are principal therapeutic strategies for liver fibrosis.^9^, ^10^, ^11^

The Krüppel‐like factors (KLFs) are a family of transcription factors containing 17 members, which are implicated in embryogenesis, proliferation, apoptosis, differentiation and development.^12^, ^13^, ^14^ KLFs consist of three evolutionarily conserved cysteine and histamine zinc fingers (C‐terminal C_2_H_2_ DNA binding domain), which recognize and bind to CACCC motifs or GC‐rich sequences in the promoter, and mediate transactivation or transrepression of the target genes.^15^ In recent years, KLF14 has evoked significant attention. KLF14 inhibited KRAS‐mediated proliferation of pancreatic cells and induced apoptosis.^16^ Overexpression of KLF14 led to G2/M arrest, and KLF14 acted as a tumour suppressor in breast ductal carcinoma and colon cancer.^17^ In hepatocellular carcinoma cells, lncRNA DGCR5 sponged miR‐346 and upregulated KLF14, and therefore inhibiting proliferation and migration.^18^ Similarly, lncRNA HAND2‐AS1 sponged miR‐1275, upregulated KLF14 expression, and therefore inhibiting proliferation and invasion of colorectal cancer cells.^19^ In summary, these studies suggested that KLF14 mainly exerted inhibitory roles in proliferation, migration and survival. Furthermore, KLF14 has recently emerged as a master regulator of multiple metabolic phenotypes in adipose tissue,^20^ and it is closely correlated with type 2 diabetes and obesity.^21^, ^22^ In addition, KLF14 not only activates the generation of lipid‐mediated signalling molecules,^23^ but also mediates lipid metabolism.^14^ Considering the vital role of KLF14 in proliferation, survival, migration and lipid metabolism, we surmised that KLF14 might regulate biological processes of HSCs, and therefore regulating liver fibrosis.

In this study, we reported that KLF14 was decreased in liver fibrosis and during HSCs activation. KLF14 overexpression increased the LD accumulation, inhibited HSCs activation, proliferation, G2/M transition, survival and migration by transactivating PPARγ. Mechanistically, KLF14 downregulation was mediated by enhancer of zeste homolog 2 (EZH2)‐regulated histone H3 lysine 27 trimethylation (H3K27me3). In animal studies, adenovirus‐mediated KLF14 overexpression ameliorated thioacetamide (TAA)‐established rat liver fibrosis through activating PPARγ signalling. Furthermore, in vivo administration of EPZ‐6438, a specific inhibitor for EZH2, dramatically alleviated TAA‐established liver fibrosis in rats. Importantly, KLF14 expression was dramatically decreased in human with fibrosis, which was significantly correlated with EZH2 upregulation and PPARγ downregulation. Collectively, this study uncovers a novel mechanism underlying liver fibrogenesis, which might contribute to better anti‐fibrotic therapies.

## 2 MATERIALS AND METHODS

### 2.1 Reagents

The PPARγ antagonist GW9662 (HY‐16578), DMNTs inhibitor 5‐azadC (HY‐A0004), EZH2 inhibitor EPZ‐6438 (HY‐13803), G9a inhibitor UNC0642 (HY‐13980) and pan‐HDAC inhibitor ITF‐2357 (HY‐14842) were purchased from MedChem Express (New Jersey, NJ, USA). All reagents were used following the standard protocols.

### 2.2 Statistical analyses

The Student's t test or one‐way ANOVA were performed to analyse data (presented as mean ± SD) using the Prism 5.0 GraphPad Software (La Jolla, CA, USA). If *P* < .05, the difference was considered statistically significant.

Detailed information of other materials and methods are provided in the Supplementary Materials.

## 3 RESULTS

### 3.1 KLF14 is inversely correlated with liver fibrosis and HSCs activation

**FIGURE 1:**
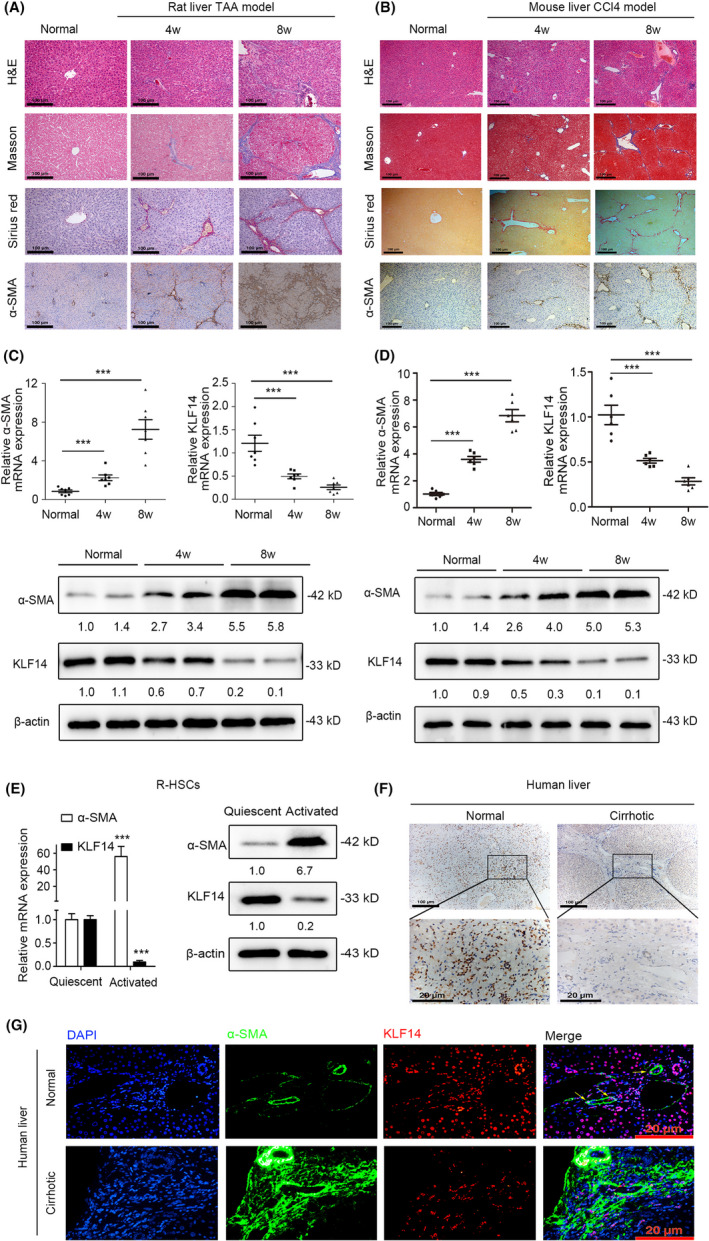
KLF14 is inversely correlated with liver fibrosis and HSCs activation. A, H&E, Masson's trichrome, Sirius red and α‐SMA staining of normal and TAA‐treated rat liver tissues (n = 7, Scale bars: 100 μm). B, H&E, Masson's trichrome, Sirius red and α‐SMA staining of normal and CCl4‐treated mouse liver tissues (n = 6, Scale bars: 100 μm). C, The normal and TAA‐treated rat liver tissues were subjected to RT‐qPCR (n = 7) and Western blotting (n = 2) analyses for detection of α‐SMA and KLF14 expression. D, The normal and CCl4‐treated mouse liver tissues were subjected to RT‐qPCR (n = 6) and Western blotting (n = 2) analyses for detection of α‐SMA and KLF14 expression. E, The quiescent and activated R‐HSCs were subjected to RT‐qPCR and Western blotting analyses for detection of α‐SMA and KLF14 expression (n = 3). F, Immunohistochemistry staining of KLF14 in human liver tissues (normal and cirrhotic, n = 3, Scale bars: 100 and 20 μm). G, Immunofluorescence staining of KLF14 and α‐SMA in human liver tissues (normal and cirrhotic, n = 3, Scale bars: 20 μm). Yellow arrows indicated HSCs. **P < .05, **P < .01, ***P < .001 versus the control group

To study the function of KLF14 in liver fibrosis, we examined KLF14 expression in TAA‐induced rat liver fibrotic model and CCl4‐induced mouse liver fibrotic model. Firstly, we assessed the degree of hepatic fibrosis in two models by haematoxylin and eosin (H&E), Masson's trichrome, Sirius red, and α‐smooth muscle actin (α‐SMA) staining. With extension of exposure to TAA or carbon tetrachloride (CCl4), the degree of the fibrosis gradually aggravated (Figure 1A,B). As the severity of liver fibrosis progressed, the mRNA and protein levels of KLF14 decreased dramatically, accompanied by upregulation of α‐SMA (Figure 1C,D).

To detect the expression of KLF14 during HSCs activation, we isolated and cultured the rat primary HSCs (R‐HSCs). The R‐HSCs cultured for 1 day were considered as quiescent HSCs, and the R‐HSCs cultured for 10 days were deemed as activated HSCs.^24^ Firstly, we measured the purity of R‐HSCs by flow cytometry analyses, which showed the purity was 97.63% (Figure S1A). The quiescent HSCs had obvious blue‐green autofluorescence under ultraviolet excitation, and they were positive for Desmin immunofluorescence staining (Figure S1B). Furthermore, the activated R‐HSCs were positive for α‐SMA immunofluorescence staining (Figure S1C). These results indicated the purity of R‐HSCs was appropriate for cellular and molecular studies. Then, we found that α‐SMA expression increased dramatically in activated HSCs, while KLF14 expression decreased significantly (Figure 1E).

Immunohistochemistry staining showed that KLF14 expression was lower in human cirrhotic liver tissues than normal liver tissues, and KLF14 was localized in the nucleus (Figure 1F, Figure S2A). Furthermore, the results of double immunofluorescence staining confirmed that KLF14 was obviously decreased in human cirrhotic liver tissues, and KLF14 was expressed both in hepatocytes and HSCs (α‐SMA positive cells) (Figure 1G, Figure S2B). Collectively, KLF14 expression is inversely correlated with liver fibrosis and HSCs activation, which might play a role in liver fibrogenesis.

### 3.2 KLF14 overexpression promotes lipid droplets accumulation in HSCs and inhibits HSCs activation

**FIGURE 2:**
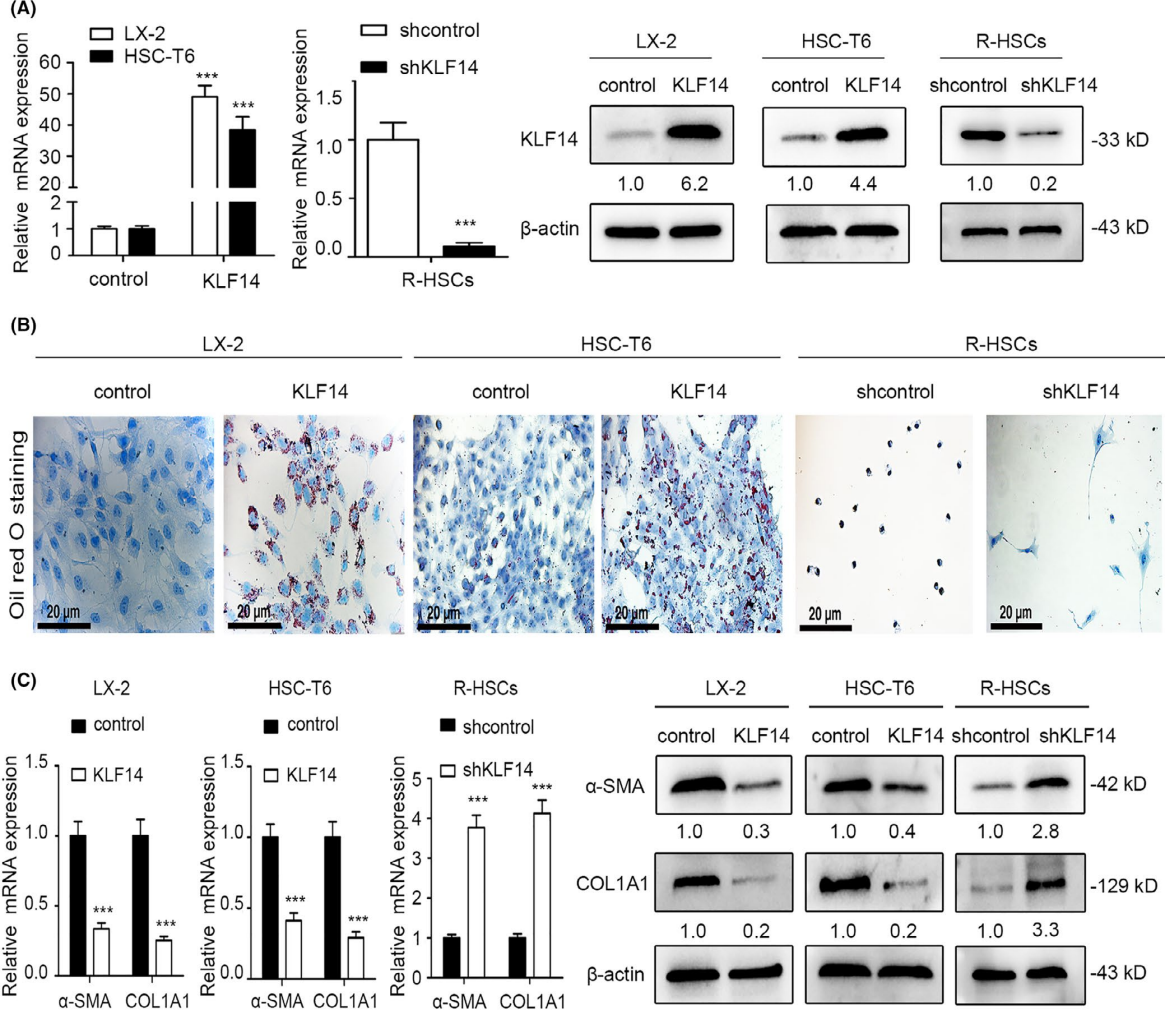
KLF14 overexpression promotes lipid droplets accumulation in HSCs and inhibits HSCs activation. A, KLF14 expression in the indicated HSCs was measured by RT‐qPCR and Western blotting analyses. B, Lipid droplets accumulation was measured by Oil Red O staining (Scale bars: 20 μm). C, Levels of α‐SMA and COL1A1 were measured by RT‐qPCR and Western blotting analyses. ***P < .001 compared with the control group. n = 3

Increasing evidences show that KLF14 is a master regulator of multiple metabolic phenotypes, especially in the lipid metabolism,^21^ and KLF14 acts as a transcriptional activator and promotes lipid generation.^23^ Thus, we are determined to explore whether KLF14 could regulate the LD content in HSCs and therefore influencing HSCs activation. To this end, we ectopically upregulated KLF14 expression by lentivirus transfection in LX‐2 and HSC‐T6 cells, and stable cell lines were established by puromycin selection. As expected, lentivirus transfection resulted in robust KLF14 overexpression (Figure 2A). Oil Red O staining results showed that KLF14 overexpression dramatically promoted the LD accumulation in both cells (Figure 2B). Furthermore, KLF14 overexpression decreased the α‐SMA and collagen A1 (COL1A1) levels (Figure 2C). In addition, we utilized the freshly isolated R‐HSCs, which were quiescent and KLF14 expression was at the highest level. By lentivirus transfection, KLF14 expression was silenced in quiescent R‐HSCs (Figure 2A). Oil Red O staining results showed that KLF14 silencing facilitated the process of LDs disappearance in R‐HSCs, and the changes in cell shape of R‐HSCs also indicated that KLF14 silencing promoted transdifferentiation of R‐HSCs (Figure 2B). Furthermore, KLF14 silencing promoted α‐SMA and COL1A1 expression (Figure 2C). Thus, KLF14 overexpression promotes LD accumulation and inhibits HSCs activation.

### 3.3 KLF14 overexpression inhibits HSCs proliferation and migration, and induces G2/M arrest and apoptosis

**FIGURE 3:**
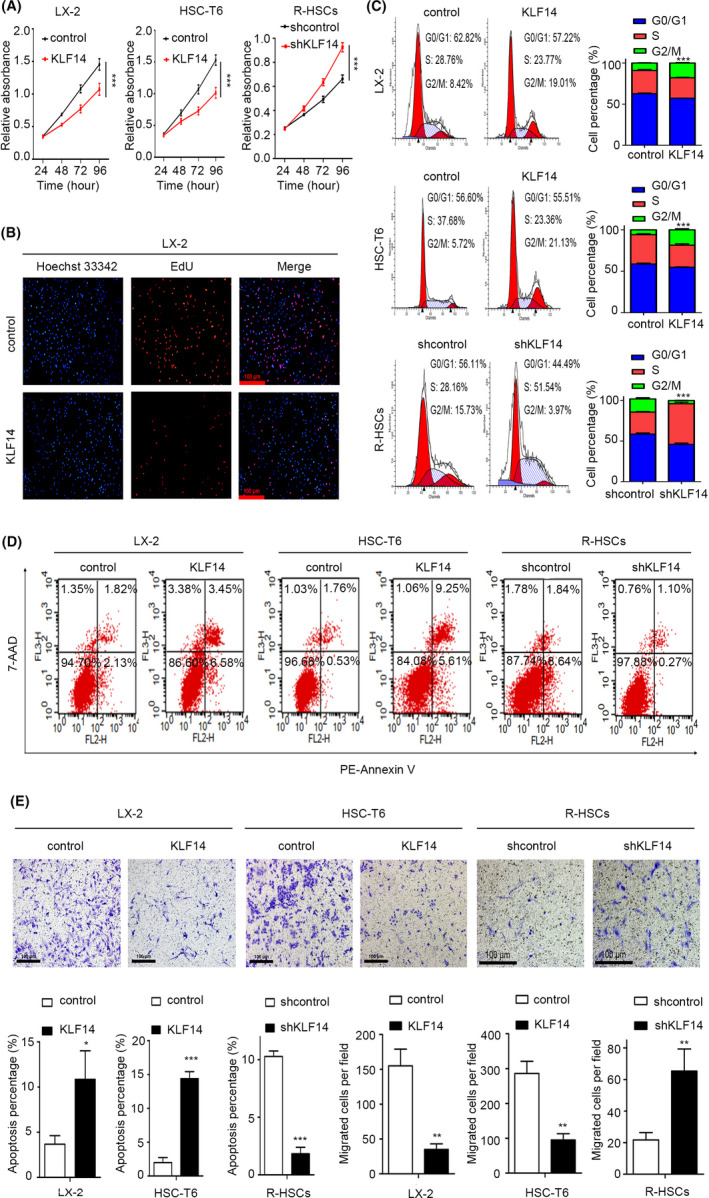
KLF14 overexpression inhibits HSCs proliferation and migration, and induces G2/M arrest and apoptosis. (A,B) Cell proliferation was assessed by CCK‐8 and EdU incorporation assays (Scale bars: 100 μm). (C,D) Cell cycle and cell apoptosis were detected by flow cytometry. E, Cell migration ability was measured by Transwell migration assay (Scale bars: 100 μm). **P < .01, ***P < .001 versus the control group. n = 3

Previous studies reported that KLF14 could regulate cell proliferation, cell cycle transition, survival and chemotaxis of several cells.^16^, ^17^, ^19^ Thus, we aimed to investigate whether KLF14 regulated these processes of HSCs. By performing Cell Counting Kit‐8 (CCK‐8) analysis, we found that KLF14 overexpression decreased the proliferation capability of LX‐2 and HSC‐T6 cells (Figure 3A), and this result was further confirmed by 5‐ethynyl‐2’‐deoxyuridine (EdU) staining in LX‐2 cells (Figure 3B). Flow cytometry analyses showed that KLF14 overexpression caused substantial G2/M phase arrest and apoptosis in both cells (Figure 3C,D). Furthermore, KLF14 overexpression decreased the migratory capability of LX‐2 and HSC‐T6 cells (Figure 3E). In addition, the inhibitory effects of KLF14 on proliferation and migration were confirmed in activated R‐HSCs (Figure S3A‐C). Furthermore, KLF14 silencing in quiescent R‐HSCs upregulated the proliferation capability, promoted cell cycle transition, decreased cell apoptosis percentage and enhanced migratory capability (Figure 3A,C‐E). Taken together, KLF14 overexpression inhibits HSCs proliferation and migration, and induced G2/M arrest and cell apoptosis.

### 3.4 KLF14 transactivates the adipogenic gene PPARγ expression in HSCs

**FIGURE 4:**
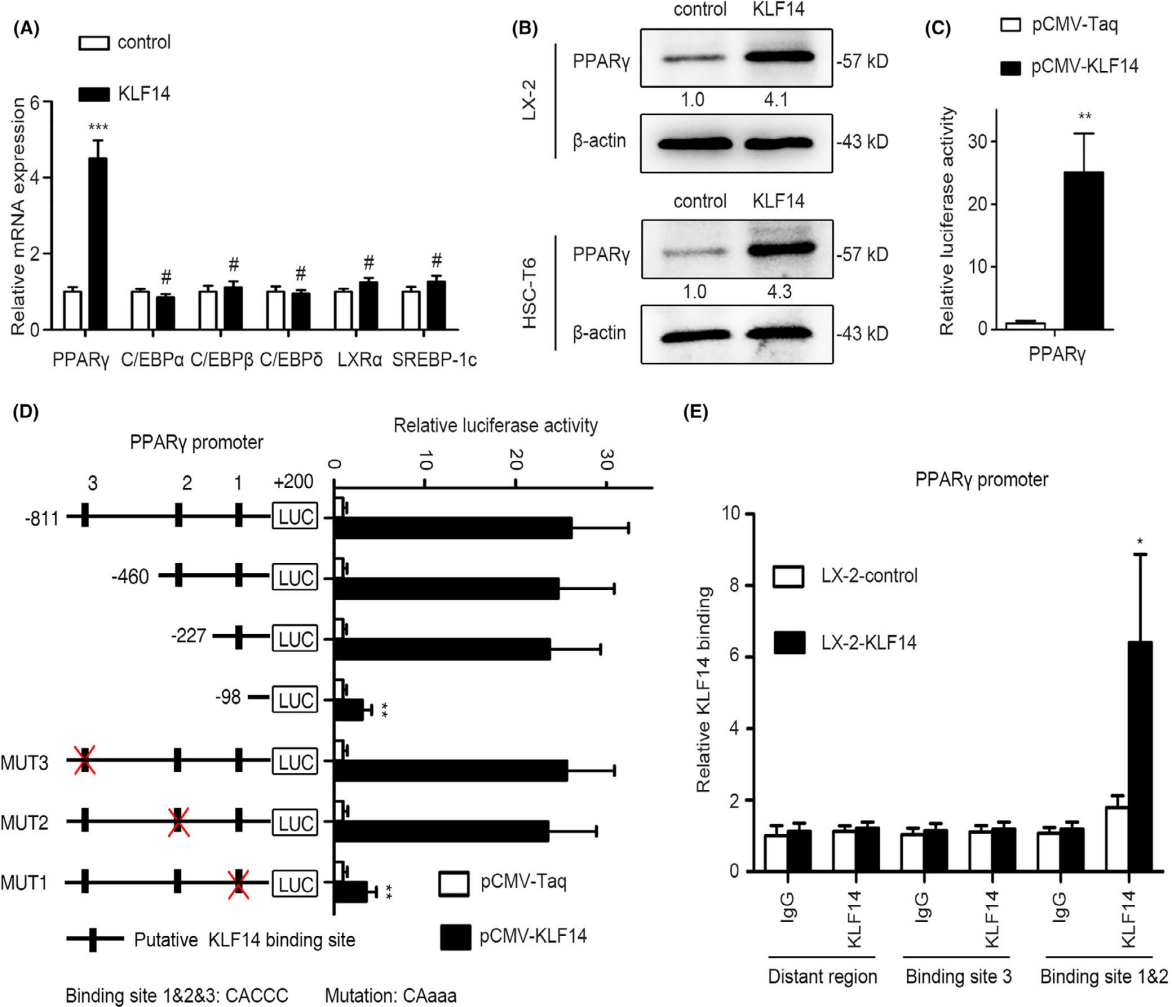
KLF14 transactivates the adipogenic gene PPARγ expression in HSCs. A, The mRNA levels of PPARγ, C/EBPα, C/EBPβ, C/EBPδ, LXRα and SREBP‐1c were measured by RT‐qPCR analysis. B, The protein level of PPARγ was assessed by Western blotting analysis. C, Luciferase reporter assays of indicated LX‐2 cells co‐transfected with pCMV‐KLF14 and PPARγ promoter luciferase construct. D, Luciferase reporter assays of indicated LX‐2 cells co‐transfected with pCMV‐KLF14 and serially truncated or mutant PPARγ promoter luciferase constructs. E, ChIP‐qPCR assay was performed to assess the direct binding of KLF14 to the PPARγ promoter in LX‐2 cells. *P < .05, **P < .01, ***P < .001, ^#^ *P* > .05 versus the control group. n = 3

Maintenance of the quiescent phenotype of HSCs is mediated by adipogenic transcription factors, including PPARγ, C/EBPs, LXRα and SREBP‐1c.^6^, ^7^ As KLF14 overexpression promoted the LD accumulation in the activated HSCs and converted activated HSCs to the quiescent phenotype, we aimed to investigate whether these effects were achieved by modulating the expression of adipogenic genes. Our data showed that KLF14 overexpression significantly promoted PPARγ mRNA expression, however, mRNA levels of C/EBPα, C/EBPβ, C/EBPδ, LXRα and SREBP‐1c were not significantly changed (Figure 4A, Figure S4A), which was validated by Western blotting analysis (Figure 4B). By luciferase reporter assay, we found that KLF14 overexpression enhanced PPARγ promoter activity, suggesting that KLF14 might transactivate PPARγ expression (Figure 4C). By sequence analysis, we identified three putative KLF14 binding motifs in the promoter region of PPARγ gene. To determine the specific binding site of KLF14 in the PPARγ promoter, a series of truncated reporters were generated with different deletions in the 5’‐flanking region of PPARγ gene, and detected their reaction to KLF14 overexpression in LX‐2 cells. The luciferase reporter assay showed that deleting the sequence between −227 and −98 bp significantly decreased the PPARγ promoter activity enhanced by KLF14 overexpression. Consistently, mutation of the putative KLF14 binding site located in this region dramatically downregulated the KLF14‐induced PPARγ promoter transactivation (Figure 4D). Furthermore, the chromatin immunoprecipitation (ChIP) assay confirmed that KLF14 binded directly to PPARγ promoter in LX‐2 cells (Figure 4E). Collectively, these data indicate that KLF14 transactivates the adipogenic gene PPARγ expression in HSCs.

### 3.5 PPARγ is essential for the inhibitory role of KLF14 overexpression in HSCs

**FIGURE 5:**
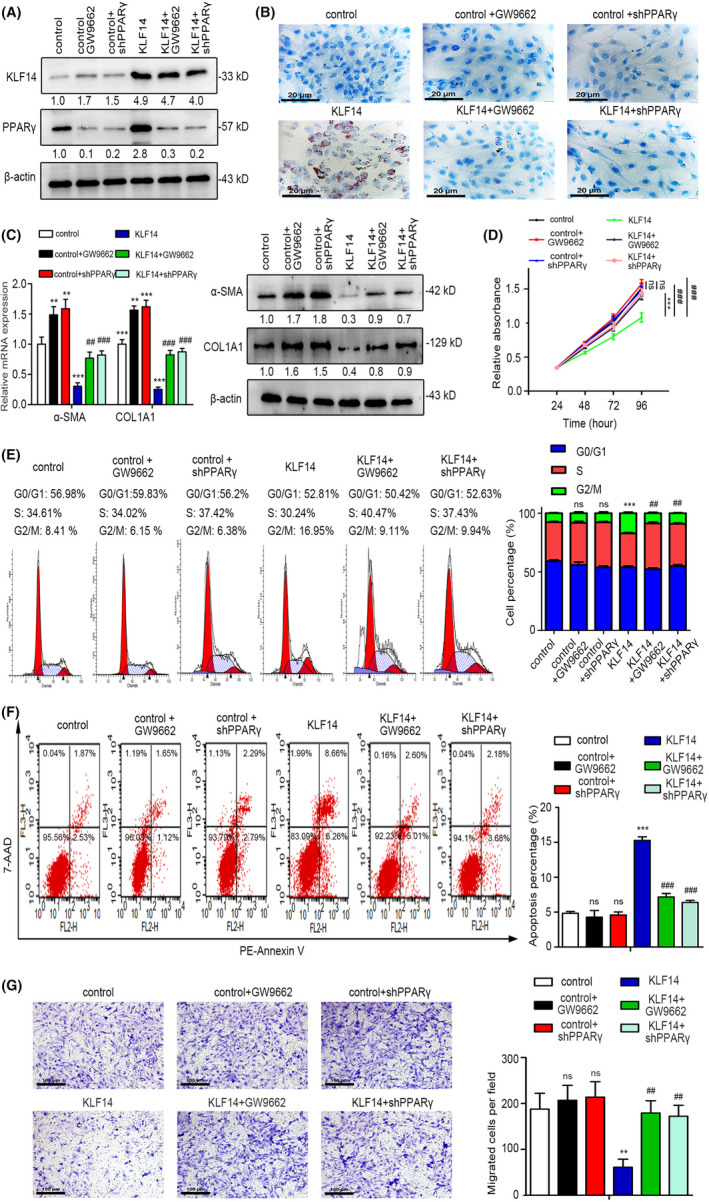
PPARγ is essential for the inhibitory role of KLF14 overexpression in HSCs. A, For PPARγ inhibition, the LX‐2‐control and LX‐2‐KLF14 cells were treated with GW9662 (1 μmol/L) for 24 h or stably transfected with LV‐shPPARγ. The expression of KLF14 and PPARγ was assessed by Western blotting analysis. B, Lipid droplets accumulation was measured by Oil Red O staining (Scale bars: 20 μm). C, Expression of α‐SMA and COL1A1 was measured by RT‐qPCR and Western blotting analyses. D, Cell proliferation was assessed by CCK‐8 assay. (E,F) Cell cycle and cell apoptosis were detected by flow cytometry. G, Cell migration ability was measured by Transwell migration assay (Scale bars: 100 μm). **P < .01, ***P < .001, ns, *P* > .05 versus the control group. ^##^ *P* < .01, ^###^ *P* < .001 versus the KLF14 group. n = 3

Previous studies reported that PPARγ was essential for LD synthesis, and it inhibited HSCs activation, proliferation, migration and induced apoptosis and senescence.^25^, ^26^, ^27^, ^28^, ^29^, ^30^ Considering PPARγ was transactivated by KLF14 in HSCs, we aimed to investigate whether KLF14 exerted its roles in PPARγ‐dependent manner. To this end, we suppressed the PPARγ signalling in stable KLF14‐overexpressing LX‐2 cells (LX‐2‐KLF14) and relevant control cells (LX‐2‐control) by selective antagonist GW9662 administration or lentivirus‐shPPARγ (LV‐shPPARγ) transfection, respectively. The inhibition efficiency of PPARγ was evaluated by Western blotting analysis (Figure 5A). The Oil Red O staining demonstrated that inhibition of PPARγ in LX‐2 cells suppressed KLF14 overexpression‐mediated LD accumulation (Figure 5B). Furthermore, KLF14 overexpression inhibited α‐SMA and COL1A1 expression, however, this suppressive effect was attenuated as a result of PPARγ inhibition (Figure 5C), indicating that KLF14 regulated HSCs activation through PPARγ. The CCK‐8 results showed that PPARγ inhibition rescued the proliferation ability of LX‐2 cells, which was inhibited by KLF14 overexpression (Figure 5D), and similar results were observed by EdU assay (Figure S4B). By performing flow cytometry analyses, we found that PPARγ inhibition decreased the percentage of cells arrested at G2/M phase, and reduced the proportion of apoptotic cells induced by KLF14 overexpression (Figure 5E,F). In addition, PPARγ inhibition rescued the reduced migratory capability of LX‐2 cells with KLF14 overexpression (Figure 5G). Notably, GW9662 administration and LV‐shPPARγ transfection were also performed in normal LX‐2 cells (LX‐2‐control) to investigate whether PPARγ inhibition alone regulated the above‐mentioned biological events of HSCs. The results showed that GW9662 administration and LV‐shPPARγ transfection further inhibited PPARγ expression (Figure 5A), and PPARγ inhibition alone further promoted α‐SMA and COL1A1 expression (Figure 5C). However, PPARγ inhibition alone has no significant effects on LD accumulation, proliferation, cell cycle transition, apoptosis and migration of normal LX‐2 cells (Figure 5B,D‐G; Figure S4B). Thus, PPARγ is essential for the inhibitory role of KLF14 overexpression in HSCs.

### 3.6 Downregulation of KLF14 in activated HSCs is mediated by EZH2

**FIGURE 6:**
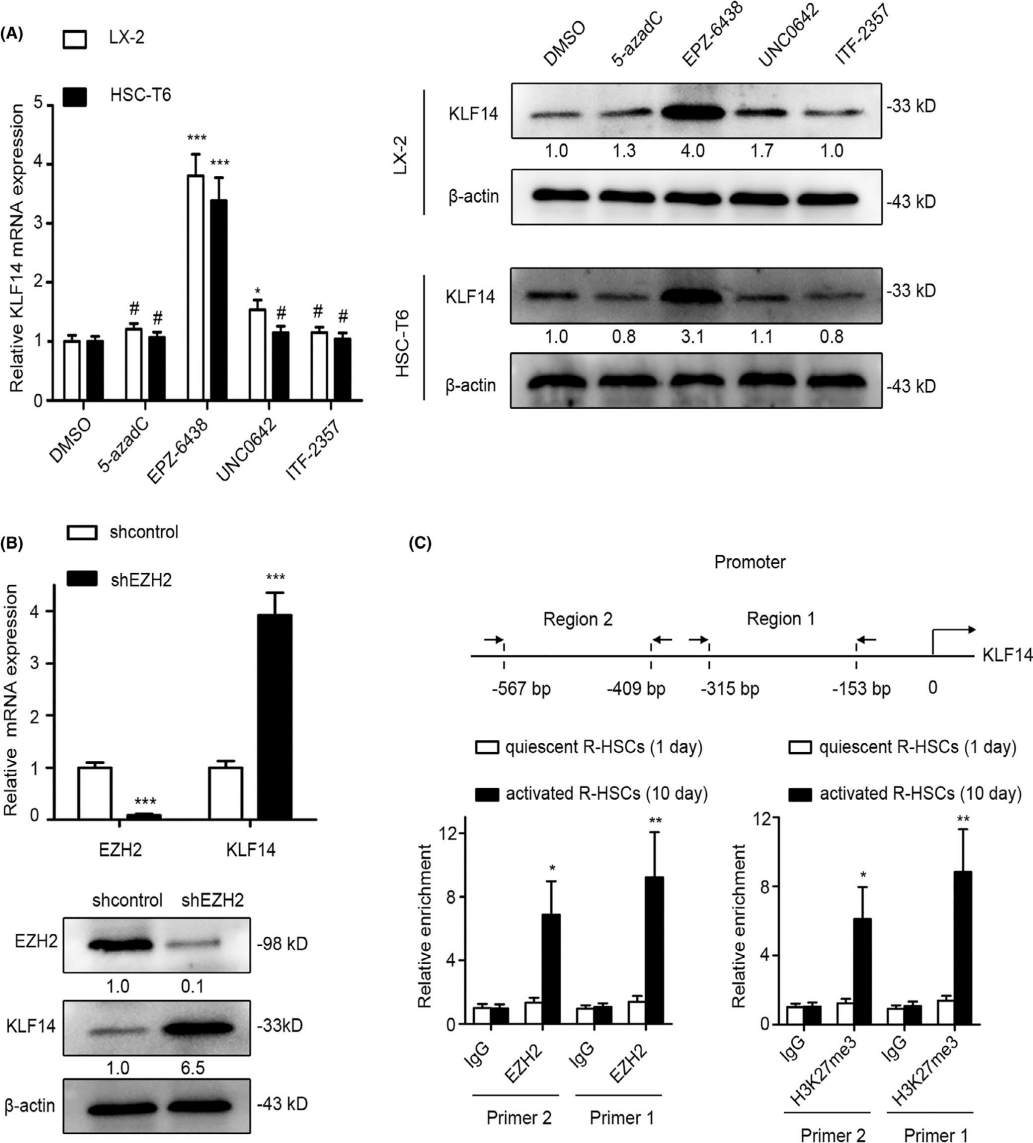
Downregulation of KLF14 in activated HSCs is mediated by EZH2. A, The indicated HSCs were subjected to DNMTs inhibitor (5‐azadC, 2 μmol/L), G9a inhibitor (UNC0642, 2 μmol/L) and pan‐HDAC inhibitor (ITF‐2357, 100 nmol/L) for 48 h, or EZH2 inhibitor (EPZ‐6438, 10 μmol/L) for 72h. KLF14 expression was measured by RT‐qPCR and Western blotting analyses. B, Expression of EZH2 and KLF14 was assessed by RT‐qPCR and Western blotting analyses. C, ChIP analysis for EZH2, H3K27me3 and IgG, and subsequent qPCR in KLF14 promoter using 2 independent primer sets in quiescent and activated R‐HSCs; IgG as a negative control. *P < .05, **P < .01, ***P < .001, ^#^ *P* > .05 versus the control group. n = 3

Epigenetic modifications are responsible for gene downregulation, such as DNA methylation, histone deacetylation and histone methylation, especially the histone H3 lysine 27 trimethylation (H3K27me3) regulated by EZH2 and histone H3 lysine 9 dimethylation regulated by the G9a, which play important roles in HSCs activation and liver fibrosis.^31^, ^32^, ^33^, ^34^ To clarify whether KLF14 downregulation in HSCs was attributed to epigenetic modification, we employed several relevant enzymes’ inhibitors, including DNA methyltransferases (DMNTs) inhibitor (5‐azadC), EZH2 inhibitor (EPZ‐6438), G9a inhibitor (UNC0642) and pan‐histone deacetylase (HDAC) inhibitor (ITF‐2357), to treat LX‐2 and HSC‐T6 cells. Among these inhibitors, EPZ‐6438 was the most powerful inducer of KLF14 mRNA and protein expression in both cells, while the role of other inhibitors was negligible or slight (Figure 6A). In addition, knockdown of endogenous EZH2 with specific LV‐shRNA led to robust reactivation of KLF14 expression in LX‐2 cells (Figure 6B). These data suggested that targeting EZH2 reactivated KLF14 expression. Furthermore, to determine whether KLF14 gene silencing in activated HSCs was directly regulated by EZH2‐mediated H3K27me3, we performed ChIP assay with antibodies against EZH2, H3K27me3 or control IgG in the quiescent and activated R‐HSCs. To assess the EZH2 occupancy and H3K27me3 modification in KLF14 promoter, the immunoprecipitated DNA samples were quantified using 2 independent primers specific for KLF14 promoter region (−567/−409 bp; −315/−153 bp). The results of ChIP‐qPCR assay showed that EZH2 was recruited to the KLF14 promoter and mediated H3K27me3 modification during HSCs activation (Figure 6C). Hence, we reveal EZH2‐regulated H3K27me3 modification is responsible for KLF14 downregulation in activated HSCs.

### 3.7 Adenovirus‐mediated KLF14 overexpression mitigates TAA‐established rat liver fibrosis through PPARγ signalling

**FIGURE 7:**
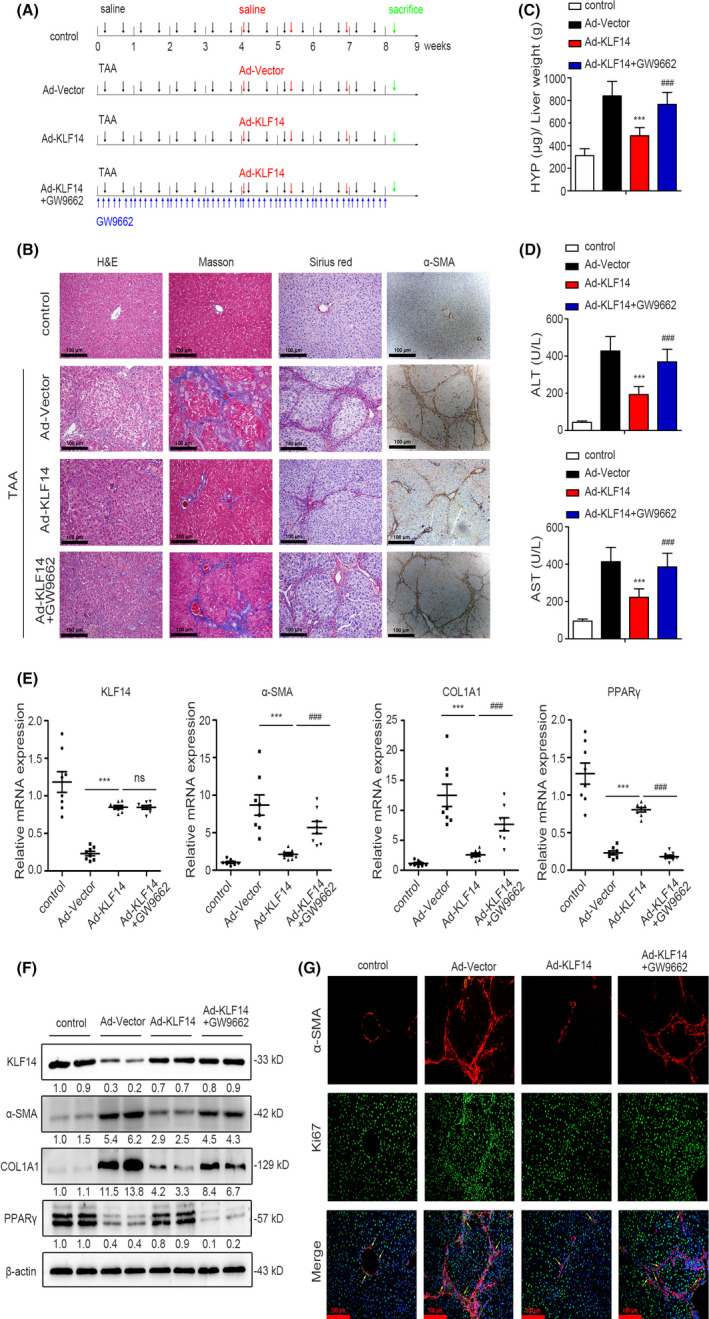
Adenovirus‐mediated KLF14 overexpression mitigates TAA‐established rat liver fibrosis through PPARγ signalling. A, Schematic diagram of the in vivo experiment, saline or TAA, Ad‐Vector or Ad‐KLF14, and GW9662 were administered accordingly (n = 8). B, H&E, Masson's trichrome, Sirius red and α‐SMA staining of rat liver sections for histological and collagen examinations (n = 8, Scale bars: 100 μm). (C,D) Liver hydroxyproline content, serum ALT and AST levels were assessed by relevant kit (n = 8). (E,F) Expression of KLF14, α‐SMA, COL1A1 and PPARγ was assessed by RT‐qPCR (n = 8) and Western blotting (n = 2) analyses. G, FITC‐conjugated Ki67antibody (green) was used to stain the proliferative cells and HSCs were presented by Cy3‐conjugated α‐SMA staining (red) (n = 3, Scale bars: 100 μm). ***P < .001 versus the Ad‐Vector group. ^###^ *P* < .001 versus the Ad‐KLF14 group. ns, *P* *> *.05

Next, we investigated whether KLF14 regulated hepatic fibrogenesis in vivo, we ectopically enforced the KLF14 expression in a TAA‐induced rat liver fibrosis model by injecting KLF14‐expressing adenovirus via tail vein, with or without PPARγ inhibition by intraperitoneal GW9662 administration (Figure 7A). The results demonstrated that TAA administration induced liver fibrosis, however, KLF14 overexpression alleviated the severity of fibrosis, which was represented by H&E, Masson's trichrome, Sirius red, and α‐SMA staining. Additionally, GW9662 administration significantly abolished the protective role of KLF14 overexpression, and aggravated the degree of fibrosis (Figure 7B). Furthermore, we evaluated the liver hydroxyproline (HYP) content and the levels of serum alanine aminotransferase (ALT) and aspartate aminotransferase (AST), and we found that KLF14 overexpression decreased the hydroxyproline content, ALT and AST levels, which were enhanced by TAA treatment, while GW9662 administration reduced the protective role of KLF14 overexpression (Figure 7C,D). In addition, KLF14 overexpression reduced TAA‐upregulated α‐SMA and COL1A1 expression, however, these effects were abolished by GW9662‐mediated PPARγ inhibition. Consistent with the in vitro results, in vivo KLF14 overexpression upregulated PPARγ expression in the fibrotic livers, however, PPARγ expression was dramatically downregulated due to GW9662 administration (Figure 7E,F). Subsequently, we assessed the proliferative HSCs in vivo by FITC‐conjugated Ki67 staining, and HSCs were characterized by Cy3‐labelled α‐SMA staining. The results showed that adenovirus‐mediated KLF14 overexpression significantly inhibited TAA‐induced HSCs proliferation, which was rescued by GW9662 administration (Figure 7G). Collectively, our study suggests that KLF14 overexpression mitigates TAA‐induced rat liver fibrosis through PPARγ signalling.

### 3.8 *In* *vivo* administration of EPZ‐6438 alleviates TAA‐induced rat liver fibrosis

**FIGURE 8:**
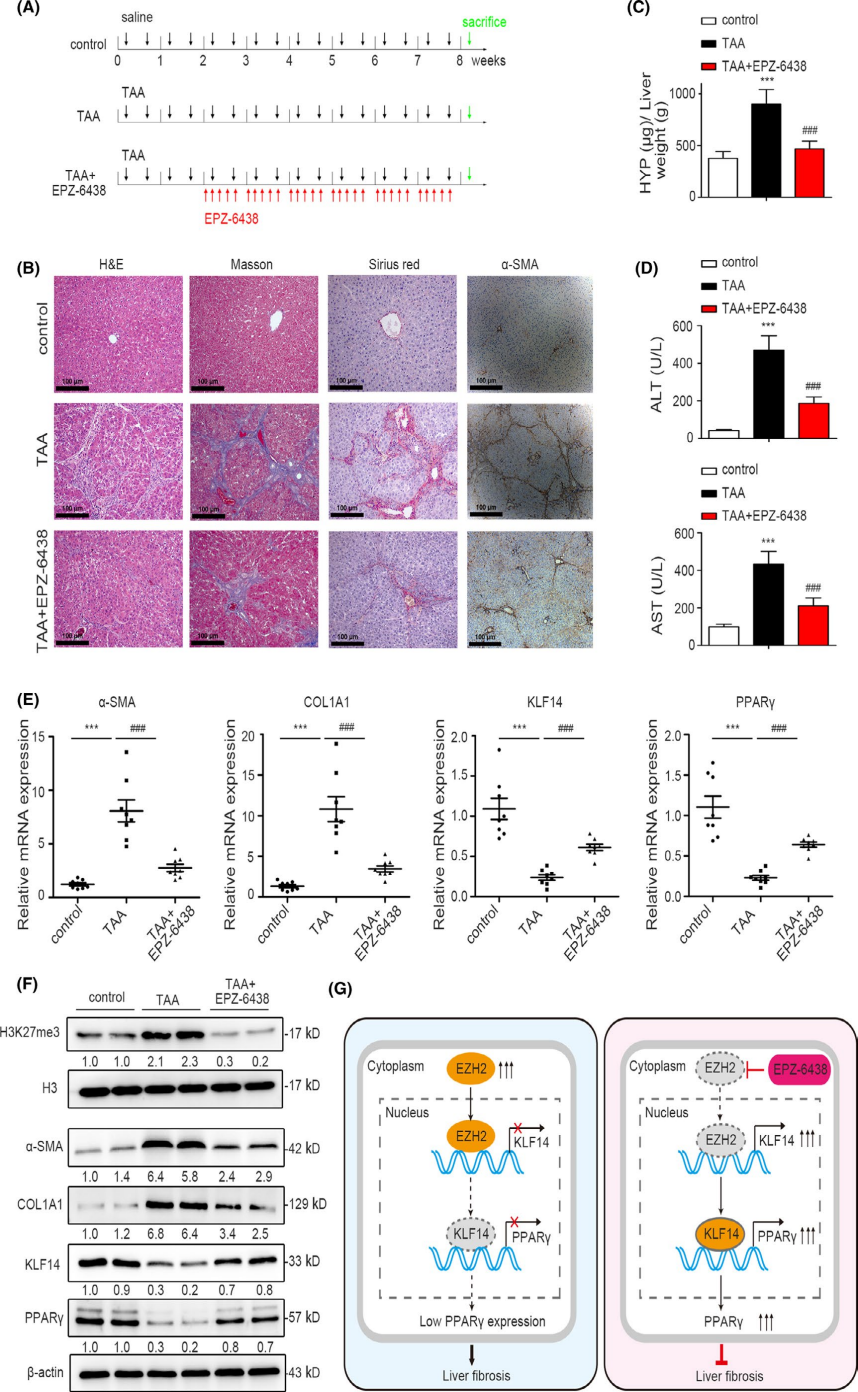
In vivo administration of EPZ‐6438 alleviates TAA‐induced rat liver fibrosis. A, Schematic diagram of the in vivo experiment, saline or TAA and EPZ‐6438 were administered accordingly (n = 8). B, H&E, Masson's trichrome, Sirius red and α‐SMA staining of rat liver sections for histological and collagen examinations (n = 8, Scale bars: 100 μm). (C,D) Liver hydroxyproline content, serum ALT and AST levels were assessed by relevant kit (n = 8). (E,F) The mRNA expression of KLF14, α‐SMA, COL1A1 and PPARγ was assessed by RT‐qPCR (n = 8). The protein expression of H3K27me3, α‐SMA, COL1A1, KLF14 and PPARγ was assessed by Western blotting analysis (n = 2). β‐actin and total H3 were used as loading control. G, A schematic diagram of the role EZH2‐KLF14‐PPARγ signalling in liver fibrosis. Upon HSCs activation, the elevated EZH2 mediates suppression of KLF14 expression, which promotes HSCs activation and liver fibrosis by downregulating PPARγ. The EZH2 inhibitor EPZ‐6438 rescues EZH2‐mediated KLF14 downregulation, which transactivates PPARγ expression, converts the activated HSCs to the quiescent phenotype and induces apoptosis, and therefore alleviating liver fibrosis. ***P < .001 versus the control group. ^###^ *P* < .001 versus the TAA group

Recently, EPZ‐6438 has been approved by FDA as the first EZH2 inhibitor for the treatment of epithelioid sarcoma,^35^ and EPZ‐6438 treatment inhibited the progression of several cancers.^36^, ^37^, ^38^ Considering KLF14 overexpression inhibited liver fibrosis in vitro and in vivo, and KLF14 expression was robustly reactivated upon EPZ‐6438 in vitro treatment, we aimed to investigate whether in vivo administration of EPZ‐6438 affected the progression of liver fibrosis. The rat fibrotic model was established by TAA injection, and EPZ‐6438 was orally administered accordingly in group Ⅲ (Figure 8A). Our data demonstrated that EPZ‐6438 administration mitigated the severity of TAA‐induced hepatic fibrosis, as represented by H&E, Masson's trichrome, Sirius red staining, as well as α‐SMA immunostaining (Figure 8B). Additionally, the hydroxyproline content, serum ALT and AST levels decreased significantly in fibrotic rats following EPZ‐6438 treatment (Figure 8C,D). Western blotting showed that EPZ‐6438 treatment decreased H3K27me3 level which was enhanced by TAA, as expected. Furthermore, EPZ‐6438 treatment reduced TAA‐induced α‐SMA and COL1A1 expression, while EPZ‐6438 treatment increased TAA‐decreased KLF14 and PPARγ expression (Figure 8E,F). Taken together, these results demonstrated that EZH2 inhibitor EPZ‐6438 upregulated KLF14 expression and ameliorated TAA‐induced rat liver fibrosis (Figure 8G).

### 3.9 KLF14 expression is significantly correlated with PPARγ and EZH2 expression in patients with liver fibrosis

**FIGURE 9:**
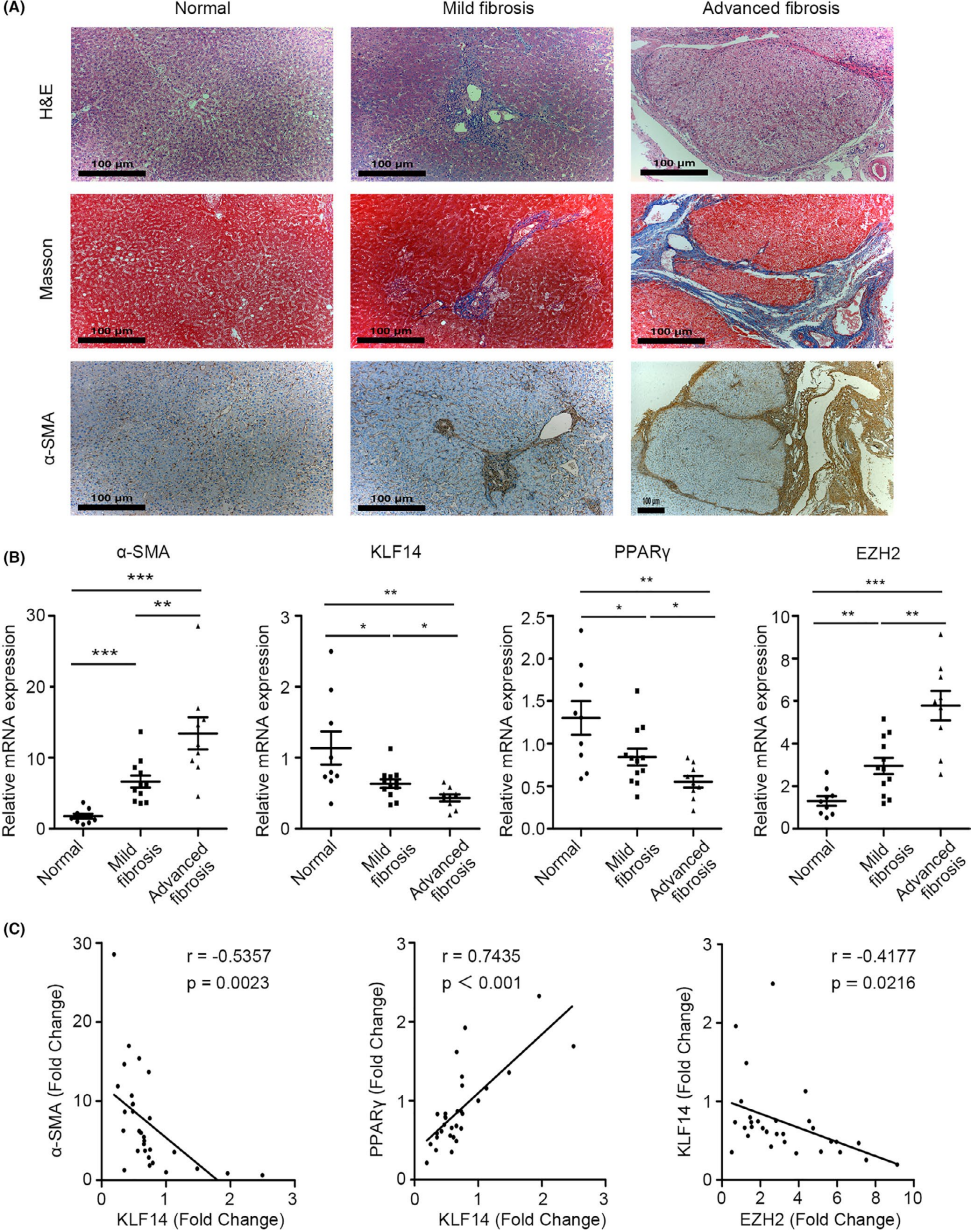
KLF14 expression is significantly correlated with PPARγ and EZH2 expression in patients with liver fibrosis. A, H&E, Masson's trichrome, and α‐SMA staining of human liver tissues from normal (n = 9), mild fibrosis (n = 12) and advanced fibrosis (n = 9). Scale bars: 100 μm. B, RT‐qPCR analyses of α‐SMA, KLF14, PPARγ and EZH2 in human liver tissues (n = 30). C, The correlations of α‐SMA, KLF14, PPARγ and EZH2 were analysed by Pearson correlation analysis (n = 30). *P < .05, **P < .01, ***P < .001 versus the control group

To address the clinical relevance, we analysed the expression of α‐SMA, KLF14, PPARγ and EZH2 in 30 human liver samples. Firstly, the human hepatic fibrosis was stratified as normal liver (n = 9), mild fibrosis (F1‐F2, n = 12), and advanced fibrosis (F3‐F4, n = 9), by H&E, Masson's trichrome staining, and α‐SMA staining (Figure 9A). Subsequently, we found that the expression of KLF14 and PPARγ was dramatically decreased in fibrotic livers, and was further downregulated with the progression of fibrosis. On the contrary, the expression of α‐SMA and EZH2 was increased with the progression of fibrosis (Figure 9B). Notably, PPARγ expression was positively correlated with KLF14 expression, and KLF14 expression was negatively correlated with EZH2 and α‐SMA expression (Figure 9C), and these results were consistent with the in vitro data and animal data. Collectively, these findings suggest that KLF14 suppression due to EZH2 elevation in fibrotic liver contributes to liver fibrosis progression by downregulating PPARγ.

## 4 DISCUSSION

Reversion of activated HSCs to the vitamin‐A‐storing phenotype and induction of apoptosis are the main therapeutic strategies for liver fibrosis.^10^, ^11^ Previously, Chen et al of our laboratory briefly reported that KLF14 was downregulated in multiple chronic liver diseases, and overexpression of KLF14 exerted a protective role in immune‐mediated liver damage by inducing differentiation of regulatory T cells.^39^ Furthermore, KLF14 was involved in proliferation, survival and migration in several tumour cells, and KLF14 mainly exerted inhibitory roles in these cellular processes, and functioned as a protective factor.^16^, ^17^, ^19^ What's more, recent studies identified that KLF14 was a master regulator in lipid metabolism.^14^, ^23^ However, roles, mechanisms and implications of KLF14 in HSCs and liver fibrogenesis have never been reported. In the present study, we reported that KLF14 was dramatically decreased in human, rat and mouse fibrotic liver tissues and during R‐HSCs activation. In addition, KLF14 overexpression promoted the LD accumulation in HSCs, inhibited HSCs activation, proliferation and migration, and induced G2/M arrest and apoptosis, which were consistent with the inhibitory roles of KLF14 in the other cells. Furthermore, adenovirus‐mediated KLF14 overexpression ameliorated TAA‐induced rat liver fibrosis. In this study, we found that, apart from the downregulation in HSCs, KLF14 expression was also downregulated in hepatocytes in the fibrotic liver tissues (Figure 1F,G), and injection of adenovirus led to KLF14 overexpression both in hepatocytes and HSCs (Figure S5A), these results drive us to think whether KLF14 could also exert hepatoprotective activities, besides the directly regulatory roles in HSCs. The FITC‐conjugated TUNEL assays showed that Ad‐KLF14 ameliorated TAA‐induced apoptosis of hepatocytes (Figure S6A). Furthermore, KLF14 overexpression inhibited apoptosis of two hepatocyte cell lines (L02 and Chang liver) (Figure S6B,C). These in vivo and in vitro results suggested that KLF14 could also exert hepatoprotective activities. Collectively, these clinical and experimental evidences strongly indicated that KLF14 exerted profound protective roles in liver fibrosis, and these effects could be achieved at least through direct regulation of HSCs and hepatoprotective activities.

The adipogenic genes play crucial roles in maintaining HSCs quiescence and inhibiting HSCs activation, such as PPARγ, C/EBPs, LXRα and SREBP‐1c.^6^, ^7^ Considering KLF14 was a master regulator of lipid metabolism and was confirmed to induce HSCs deactivation, we aimed to investigate whether KLF14 exerted these effects by regulating the expression of adipogenic genes. Interestingly, we found that KLF14 dramatically upregulated PPARγ expression both in LX‐2 and HSC‐T6 cells. Mechanistically, as transcription factors, KLFs exert the transactivation or transrepression function by binding to the CACCC motifs or GC‐rich sequences in the promoter of target genes,^15^ and the role varies in different cells and diseases. KLF14 was identified as a transrepressor by the Raul Urrutia's team for the first time, which coupled to mSin3A and HDAC2, and repressed the TGF‐β receptor II promoter in pancreatic epithelial cancer cell lines.^40^ Subsequently, they found that KLF14 transrepressed Cyclin A2 promoter in pancreatic cancer cell lines.^16^ Furthermore, Fan et al reported that KLF14 transrepressed Polo‐like kinase 4 in breast ductal carcinoma and colon cancer cells.^17^ On the contrary, several studies reported the transactivating role of KLF14. For the first time, the Raul Urrutia's team found that KLF14 transactivated sphingosine kinase 1, which regulated the generation of sphingosine‐1‐phosphate, and therefore mediating endothelial cell growth, survival, differentiation and motility.^23^ Subsequently, Guo et al reported that KLF14 transactivated apolipoprotein A‐I expression and reduced atherosclerosis.^14^ In addition, KLF14 transactivated HMOX1, which encoded haem oxygenase‐1 and enhanced antioxidant properties of prostate cancer cells.^41^ Furthermore, KLF14 transactivated peroxisome proliferator‐activated receptor‐coactivator 1α and regulated hepatic gluconeogenesis in mice.^42^ These conflicting findings suggested that whether KLF14 acted as a transactivator or transrepressor in different cells needed further exploration. In the current study, we found that KLF14 directly transactivated PPARγ promoter and upregulated its expression. It has been well defined that PPARγ inhibited liver fibrogenesis. PPARγ expression was downregulated during HSCs activation, and its overexpression significantly recovered LD storage in HSCs, and inhibited activation, proliferation, contraction, adhesion, migration, and induced apoptosis and senescence of HSCs, and therefore attenuating liver fibrosis.^25^, ^26^, ^27^, ^28^, ^29^, ^30^ Furthermore, PPARγ ligand induced G2/M arrest in cholangiocarcinoma cell.^43^ By using PPARγ antagonist GW9662 and specific LV‐shRNA, we found that PPARγ inhibition significantly decreased the inhibitory roles of KLF14 overexpression in HSCs, and in vivo study confirmed that KLF14 overexpression ameliorated TAA‐induced liver fibrosis in PPARγ‐dependent manner. More importantly, there existed a strongly positive correlation between KLF14 expression and PPARγ expression in patients with liver fibrosis. Notably, our results showed that PPARγ inhibition alone further promoted activation, but have no significant effects on LD accumulation, proliferation, cell cycle transition, apoptosis and migration of normal LX‐2 cells. Our results were similar to several previous studies.^26^, ^29^, ^44^ We thought these results might be caused by the low baseline expression level of endogenous PPARγ in LX‐2 cells, thus, further inhibition of PPARγ could not augment the pro‐fibrotic role of PPARγ deficiency. Furthermore, our results demonstrated that PPARγ overexpression could not reciprocally regulate expression of KLF14 (Figure S7A). Taken together, KLF14 overexpression transactivated PPARγ, and therefore converting the activated HSCs to the quiescent phenotype, thus alleviating liver fibrosis. However, the mechanism underlying the hepatoprotective activities of KLF14 needs further exploration.

Epigenetic modifications play important roles in HSCs activation and liver fibrosis, especially the DNA methylation, histone methylation (especially H3K27me3 and H3K9me2) and histone deacetylation, which lead to aberrant transrepression state.^31^, ^32^, ^33^, ^34^ Several studies reported that, among the ageing‐related CpG sites, KLF14 promoter region was associated with chronological age.^45^, ^46^, ^47^ Furthermore, with ageing and obesity, the level of DNA methylation in KLF14 promoter was increased significantly in several organs in mice, which led to downregulation of KLF14.^47^ Moreover, the KLF14 gene was under a hypermethylation state in familial Alzheimer's disease, which regulated cell death signalling.^48^ However, in this study, we found that inhibition of DNMTs using 5‐azadC did not significantly increased KLF14 expression, however, targeting EZH2 by EPZ‐6438 or LV‐shRNA dramatically reactivated KLF14 expression in HSCs. In addition, ChIP assay confirmed the abundant binding of EZH2 and H3K27me3 on KLF14 promoter in HSCs. Furthermore, KLF14 expression was negatively correlated with EZH2 expression in patients with liver fibrosis. In addition, we found inverse expression pattern of KLF14/PPARγ and EZH2 in R‐HSCs (Figure S8A), suggesting that inverse expression of KLF14/PPARγ and EZH2 in fibrotic livers originates from HSCs. Thus, we demonstrated for the first time that the transcriptional silencing of KLF14 during HSCs activation was mediated by EZH2‐regulated H3K27me3, rather than DNMTs‐induced DNA methylation. This discrepancy might be caused by different genetic backgrounds of the cells, which needs further exploration.

Polycomb repressive complex 2 consists of the catalytic subunit EZH2, which catalyses H3K27 trimethylation and leads to repression of gene expression.^49^ Recent studies showed that EZH2 played critical role in initiation and development of various tumours, and blocking EZH2 signalling by EPZ‐6438 inhibited tumour progression.^36^, ^37^, ^38^ Strikingly, EPZ‐6438 (Tazemetostat, Tazverik™), as a first‐in‐class, small molecular inhibitor of EZH2, received accelerated approval in January 2020 by FDA for the treatment of patients (age ≥ 16 years) diagnosed with locally advanced or metastatic epithelioid sarcoma, which are ineligible for complete surgical resection.^35^ EPZ‐6438 comes to be the first approved EZH2 inhibitor for clinical treatment. It is also undergoing several other clinical trials as anticancer agent.^50^, ^51^ In this study, our data showed that in vivo administration of EPZ‐6438 dramatically alleviated TAA‐induced rat liver fibrosis, indicating that EPZ‐6438 might have a broader range of application, which is appealing and needs further clinical investigation. As previously reported, the stimulation of EZH2 expression and subsequent H3K27 trimethylation of PPARγ gene is one mechanism for MeCP2‐mediated transcriptional silencing of PPARγ.^52^ Our results confirmed EPZ‐6438 treatment reactivated PPARγ expression in LX‐2 and HSC‐T6 cells (Figure S7B). From our perspective, there might be at least three possible underlying mechanism for the suppressive effect of EPZ‐6438. Firstly, EPZ‐6438 inhibited the activity of EZH2, and rescued the expression of PPARγ, which was directly inhibited by EZH2 (direct regulation of PPARγ by EZH2). Secondly, EPZ‐6438 abolished the suppression of KLF14 by EZH2, and reactivated KLF14 transcription, which subsequently transactivated PPARγ expression (indirect regulation of PPARγ by EZH2 via KLF14). According to our results, EPZ‐6438 administration upregulated the mRNA and protein expression of PPARγ and KLF14, which were both downregulated in fibrotic liver tissues (Figure 8E,F). We thought these results could at least confirm the existence of the indirect regulation, as we have confirmed KLF14 overexpression did alleviate liver fibrosis in vitro and in vivo. Thirdly, there might exist other genes also critical for anti‐fibrosis, and these genes were transrepressed by EZH2, in this sense, EPZ‐6438 administration might result in transactivation of these anti‐fibrotic genes, and therefore alleviating liver fibrosis (the unknown way). Collectively, EPZ‐6438 could alleviate TAA‐induced liver fibrosis by directly regulated PPARγ expression and indirectly regulated PPARγ expression via KLF14. Therefore, as an intermediate molecule, KLF14 did exert a significant role in the EZH2/PPARγ axis to some extent. However, whether there exists other mechanism for the effect of EPZ‐6438 needs further exploration.

In summary, we reported a protective role for KLF14 in liver fibrosis. KLF14 reversed the activated HSCs to the quiescent phenotype and inhibited TAA‐induced liver fibrosis by transactivating PPARγ expression. EZH2‐mediated H3K27me3 modification is a novel mechanism responsible for KLF14 downregulation in HSCs activation. EPZ‐6438, a specific EZH2 inhibitor, dramatically ameliorated TAA‐induced rat hepatic fibrosis. Thus, KLF14 exerts a critical role in liver fibrogenesis, and targeting the EZH2/KLF14/PPARγ axis might provide a novel approach to liver fibrosis treatment.

## CONFLICT OF INTEREST

The authors declare that they have no conflicts of interest.

## AUTHOR CONTRIBUTIONS

ZD performed the experiments and wrote the manuscript. ML, ZW, ZL and YF helped in tissues collection and data analysis. LX, DT and ZD designed the project. LX and DT revised the manuscript.

## Supporting information

Fig S1

Fig S2

Fig S3

Fig S4

Fig S5

Fig S6

Fig S7

Fig S8

Method S1

## Data Availability

All supporting data are included in the article and its additional files.
