# Supplementary figures and images for: EZH2‐mediated inhibition of KLF14 expression promotes HSCs activation and liver fibrosis by downregulating PPARγ
Source: Cell Prolif. 2021 May 24;54(7):e13072. doi: 10.1111/cpr.13072 (PMC8249795; doi:10.1111/cpr.13072)

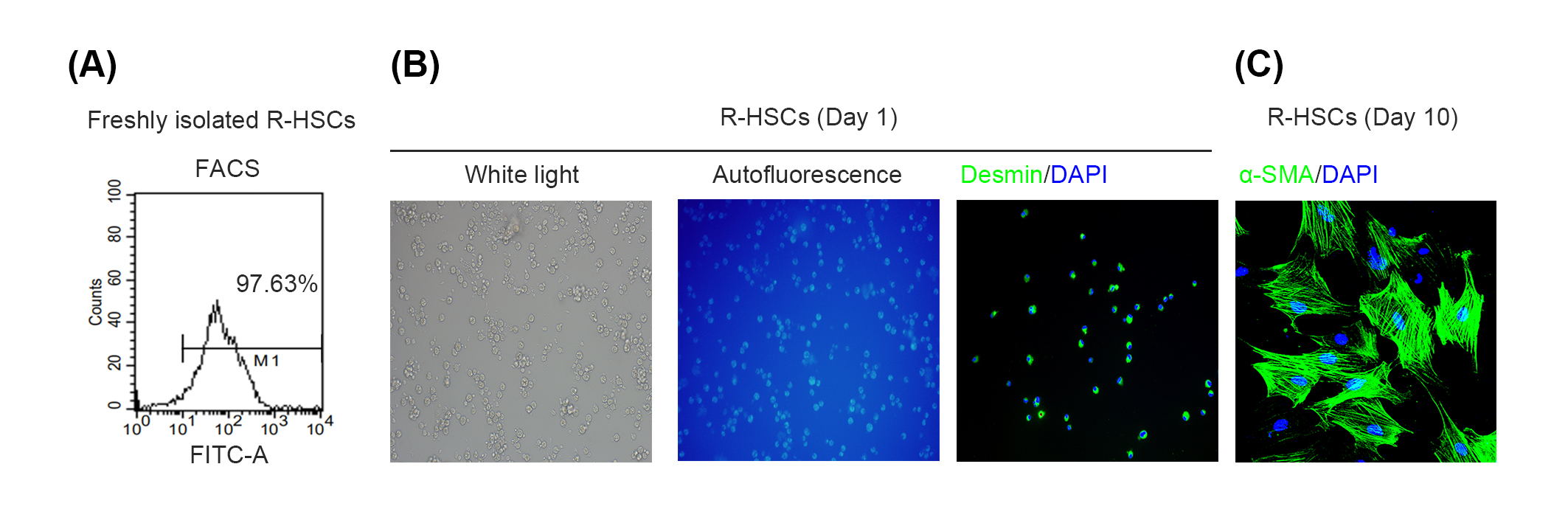

Supplement: Supplementary file 1 — Fig S1 [file CPR-54-e13072-s005.jpg]

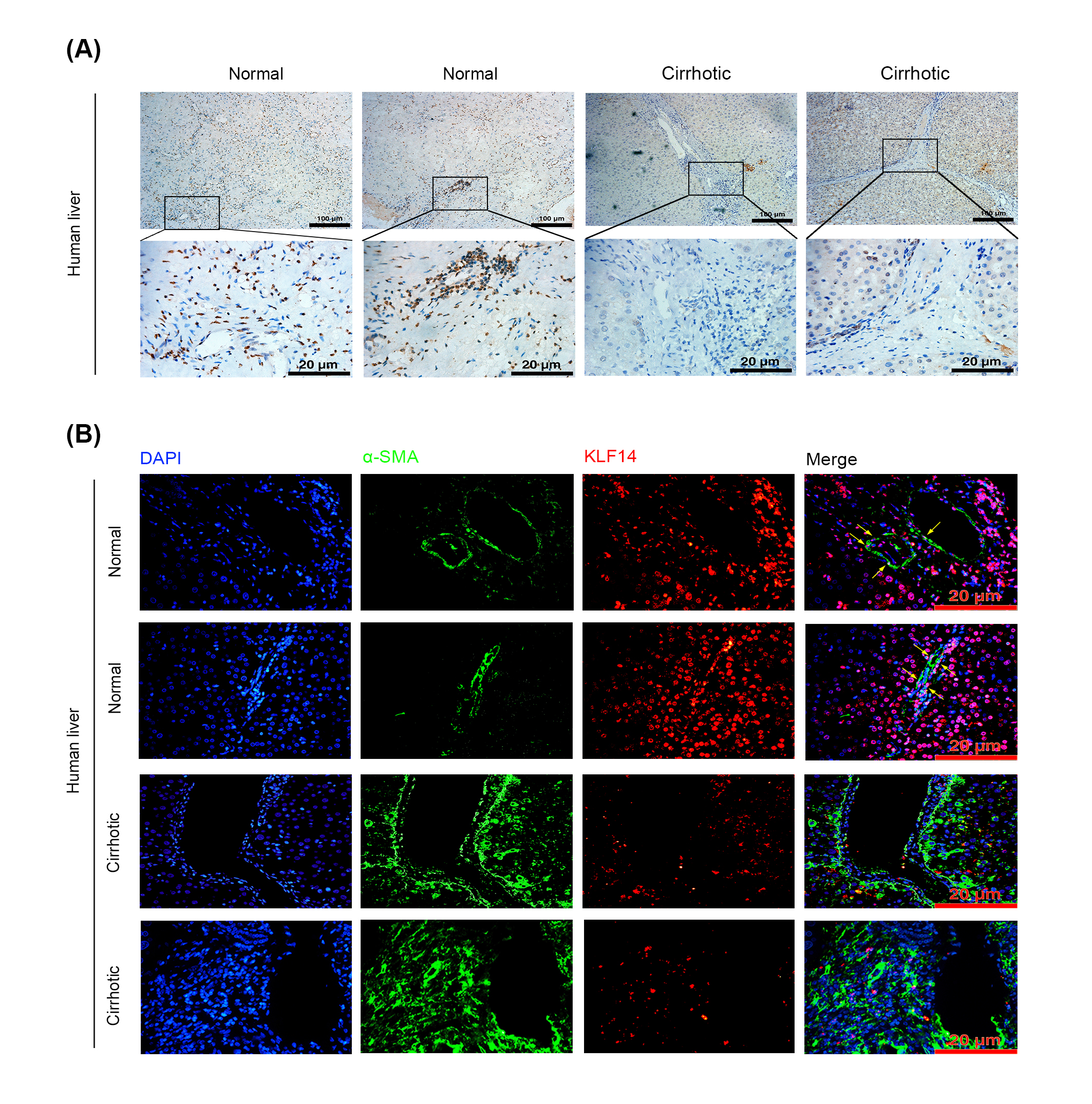

Supplement: Supplementary file 2 — Fig S2 [file CPR-54-e13072-s003.jpg]

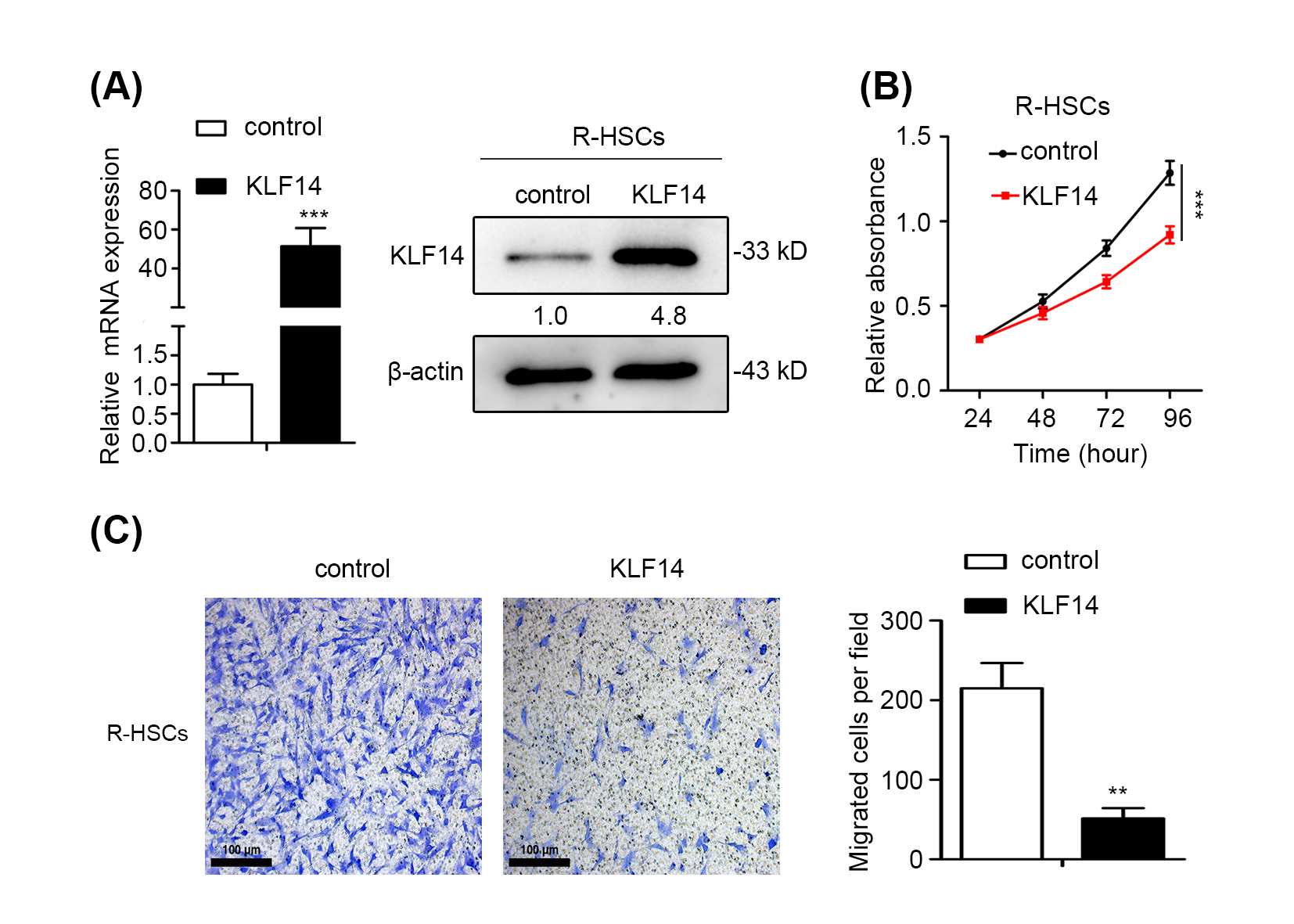

Supplement: Supplementary file 3 — Fig S3 [file CPR-54-e13072-s001.jpg]

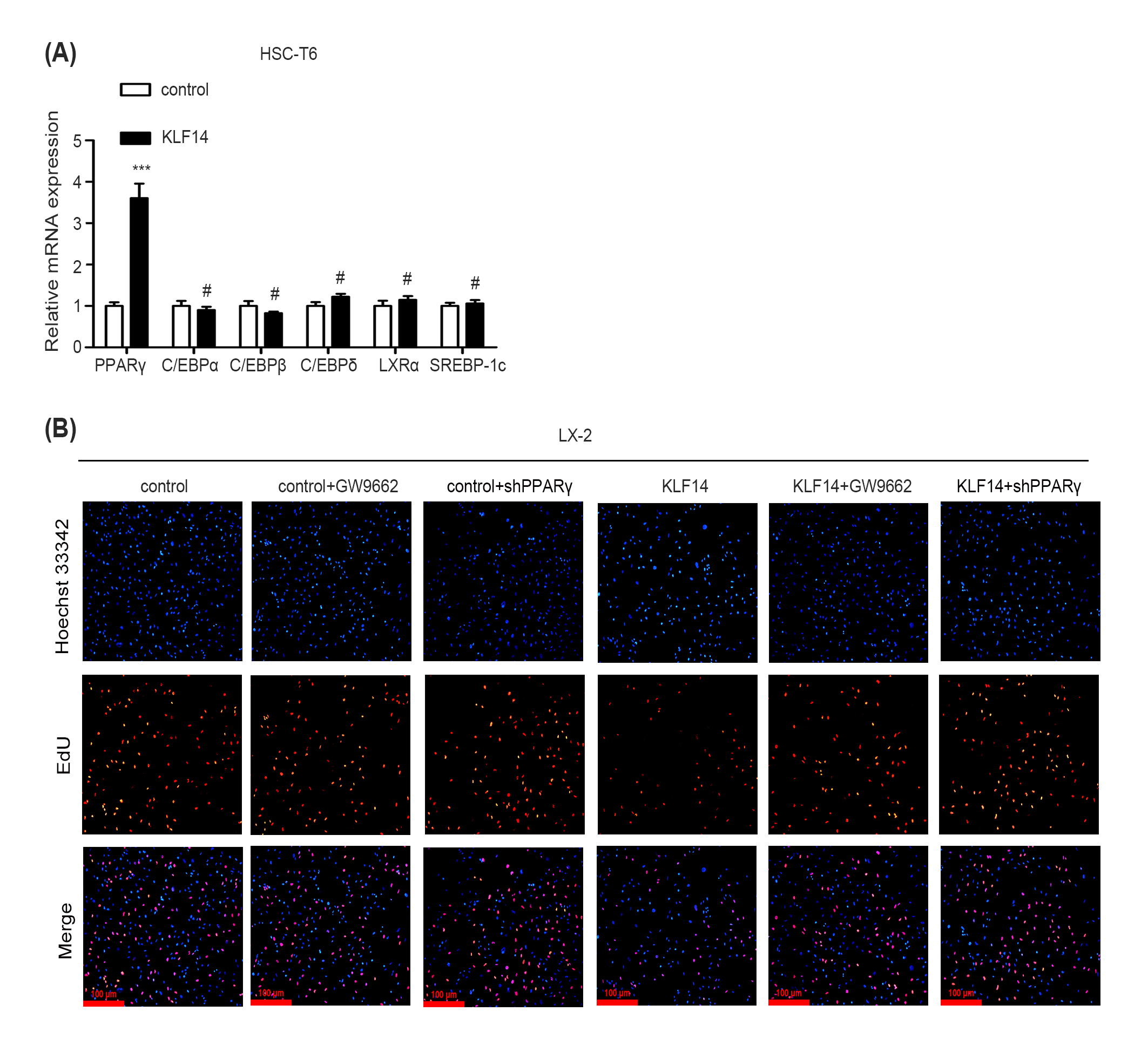

Supplement: Supplementary file 4 — Fig S4 [file CPR-54-e13072-s007.jpg]

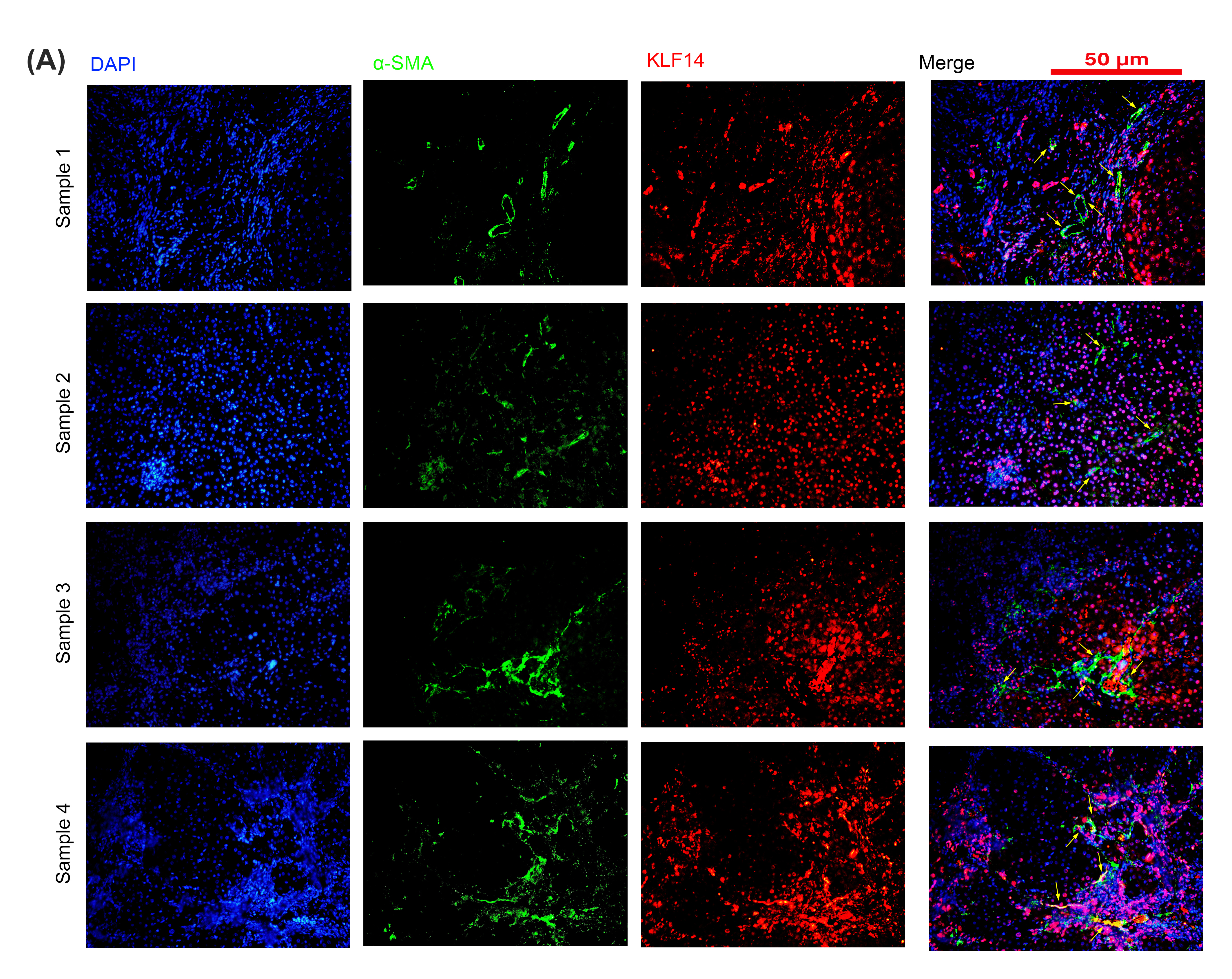

Supplement: Supplementary file 5 — Fig S5 [file CPR-54-e13072-s006.jpg]

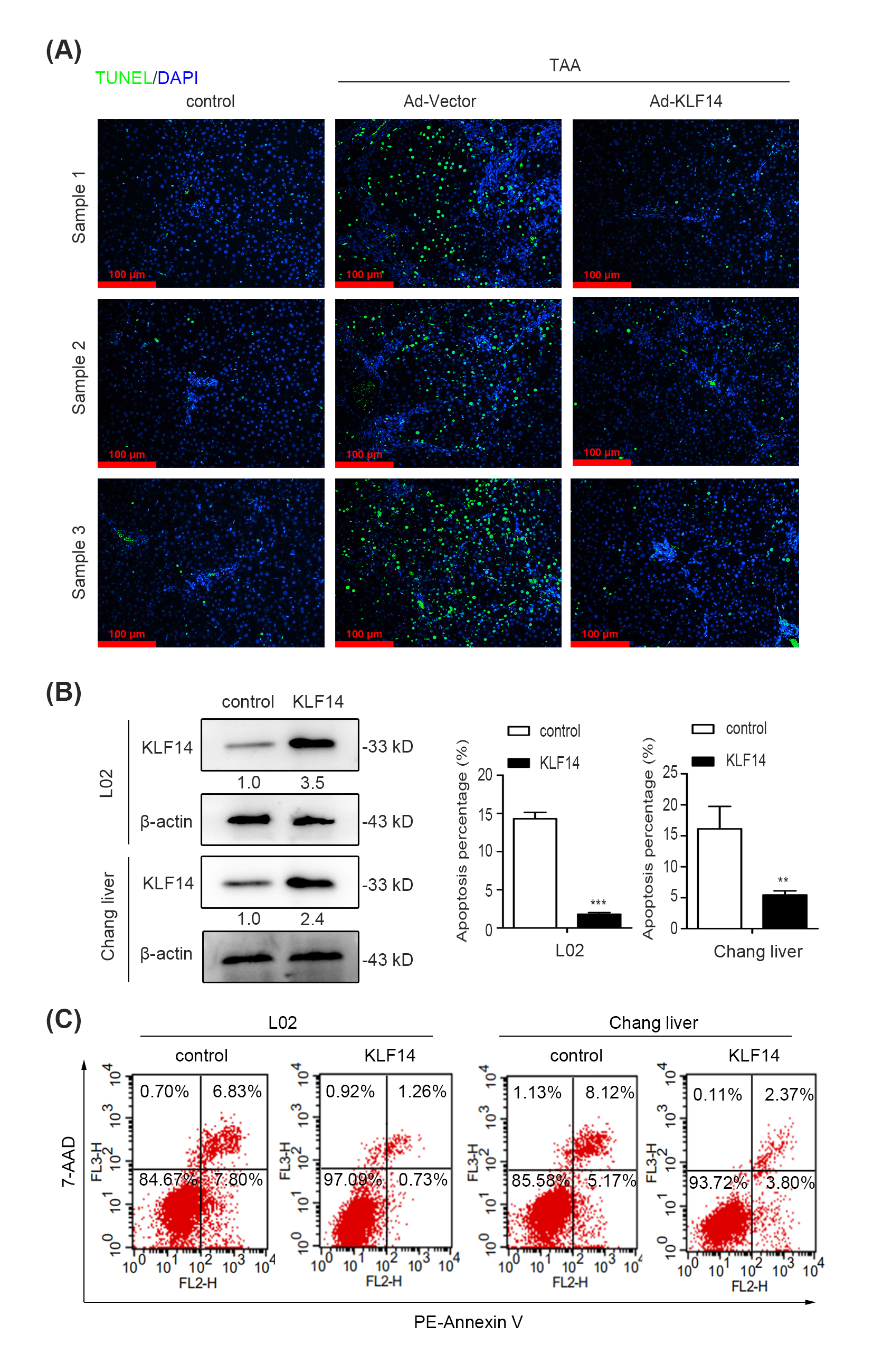

Supplement: Supplementary file 6 — Fig S6 [file CPR-54-e13072-s002.jpg]

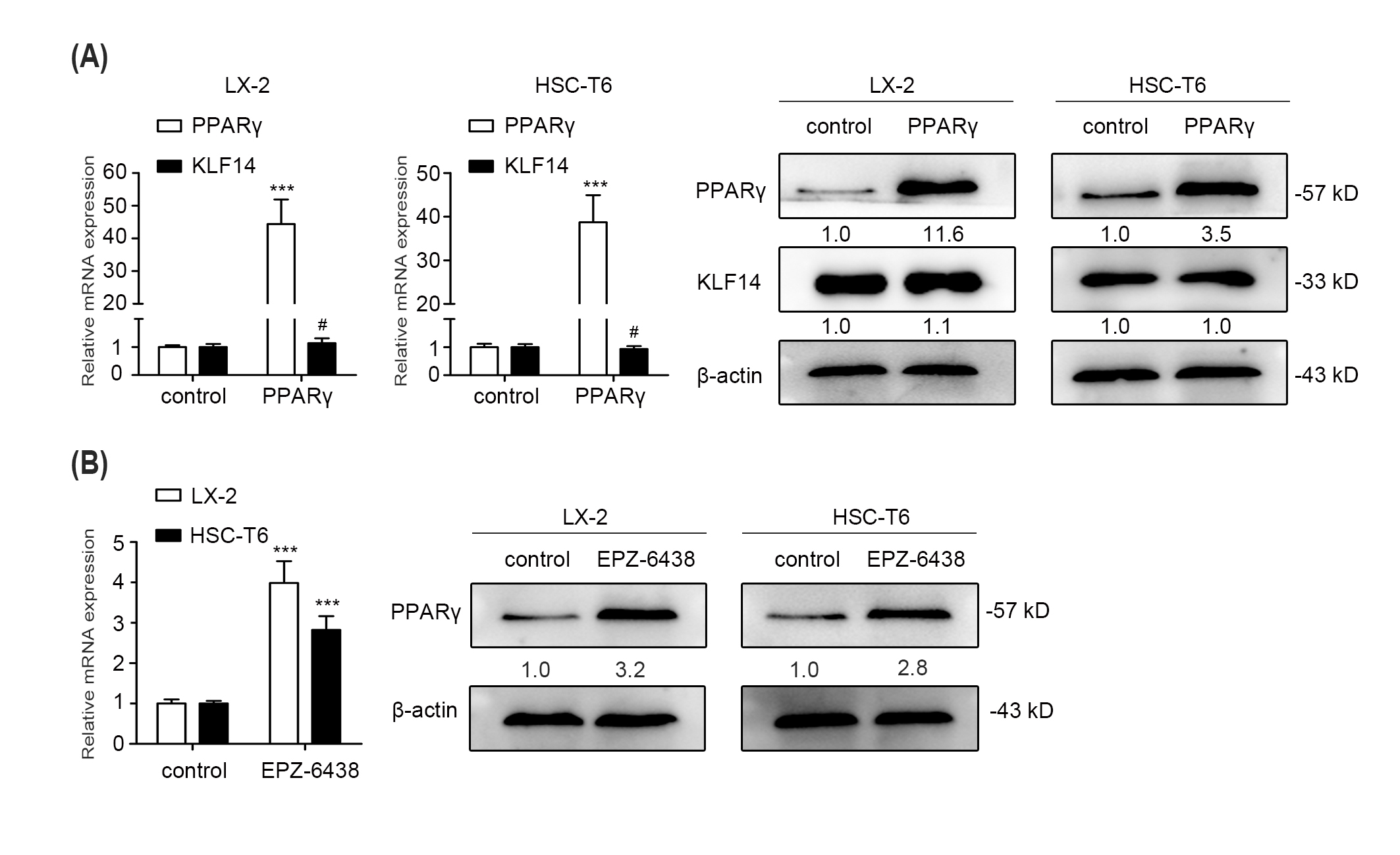

Supplement: Supplementary file 7 — Fig S7 [file CPR-54-e13072-s008.jpg]

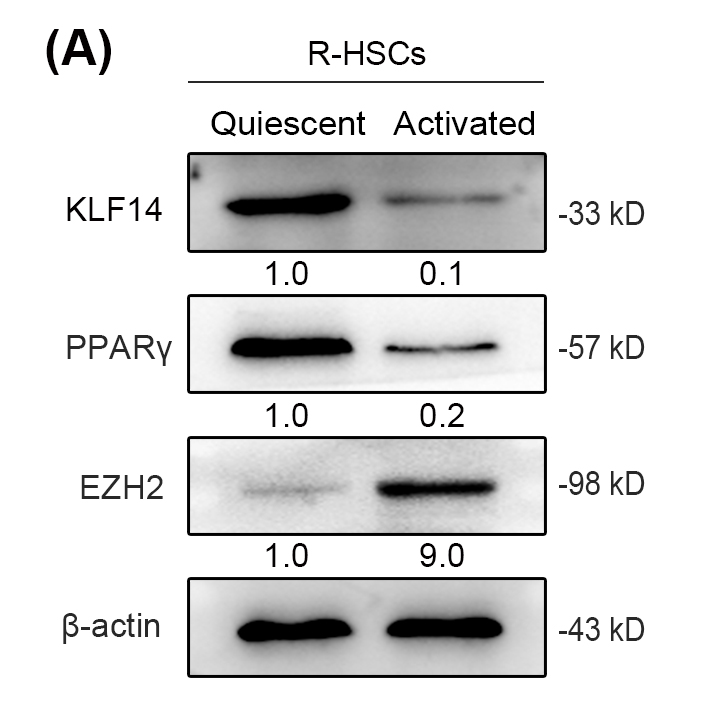

Supplement: Supplementary file 8 — Fig S8 [file CPR-54-e13072-s004.jpg]
