# Supplementary material for: EZH2‐mediated inhibition of KLF14 expression promotes HSCs activation and liver fibrosis by downregulating PPARγ
Source: Cell Prolif. 2021 May 24;54(7):e13072. doi: 10.1111/cpr.13072 (PMC8249795; doi:10.1111/cpr.13072)
Supplement: Supplementary file 9 — Method S1 [file CPR-54-e13072-s009.docx]

**Supplementary information**

**EZH2-mediated inhibition of KLF14 expression promotes HSCs activation and liver fibrosis by downregulating PPARγ**

Zhipeng Du^1,#^, Mei Liu^1,#^, Zhihui Wang^1^, Zhuoying Lin^1^, Yangyang Feng^1^, Dean Tian^1,*^, Limin Xia^1,*^

^1^Department of Gastroenterology, Institute of Liver and Gastrointestinal Diseases, Hubei Key Laboratory of Hepato-Pancreato-Biliary Diseases, Tongji Hospital of Tongji Medical College, Huazhong University of Science and Technology, Wuhan 430030, Hubei Province, China

^#^These authors contributed equally to this work.

***Corresponding author**: Dr. Limin Xia and Dr. Dean Tian, Department of Gastroenterology, Institute of Liver and Gastrointestinal Diseases, Hubei Key Laboratory of Hepato-Pancreato-Biliary Diseases, Tongji Hospital of Tongji Medical College, Huazhong University of Science and Technology, Wuhan 430030, Hubei Province, China; Phone: 86 27 6937 8507; Fax: 86 27 8366 2832; Email: [xialimin@tjh.tjmu.edu.cn](mailto:xialimin@tjh.tjmu.edu.cn), and [datian@tjh.tjmu.edu.cn](mailto:datian@tjh.tjmu.edu.cn)

**Supplementary Material and Methods**

**Human liver samples**

Written informed consent was supplied by all the patients. The study was approved by the Ethics Committee of Tongji Hospital, and it conformed to the provisions of the Declaration of Helsinki. Totally, 9 human normal liver tissues (hepatic haemangioma tissues) and 21 human fibrotic liver tissues (the paracancerous tissues from liver cancer patients) were acquired from surgical resection without preoperative treatment at Tongji Hospital of Tongji Medical College, Huazhong University of Science and Technology (HUST) (Wuhan, China). To score the degree of hepatic fibrosis, the paraffin-embedded 5 μm thick slides were used to perform hematoxylin and eosin (H&E), Masson’s trichrome, Sirius red, and α-smooth muscle actin (α-SMA) staining, and the stages (stage F0-F4) of fibrosis were scored by three pathologists blinded to the study, following the METAVIR fibrosis staging system.^[1](#_ENREF_1)^ Then, hepatic fibrosis was stratified as normal liver (F0, n=9)，mild fibrosis (F1-F2, n=12), and advanced fibrosis (F3-F4, n=9).

**Cell culture**

Isolation of R-HSCs was performed using male Sprague Dawley (SD) rats, as previously described.^[2](#_ENREF_2)^ The immortalized human HSCs cell line LX-2 was obtained from Central South University (Changsha, China) and rat HSCs cell line HSC-T6 was preserved in our institute. R-HSCs were cultivated in Dulbecco’s modified Eagle’s medium (DMEM) (KeyGen Biotech, Nanjing, China) supplemented with 20% fetal bovine serum (FBS) (Gibco, Carlsbad, CA, USA), while LX-2 and HSC-T6 cells were cultivated in DMEM containing 10% FBS.

**Animal models of liver fibrosis**

All the *in vivo* studies were conducted according to the international and national guidelines. The protocol obtained approval from the Ethics Committee of Animal Experiments of Tongji Medical College, HUST. Rats (male, about 250 g) and C57BL/6 mice (male, about 8-week-old) were obtained and raised in Department of Experimental Animals. For TAA models of liver fibrosis (n=8 each group), TAA was dissolved using saline, and then, the rats received intraperitoneal injection of TAA (150 mg/kg body weight, twice per week for 4 or 8 weeks) or an equivalent amount of saline, as previously described.^[2](#_ENREF_2)^ For CCl4 models of liver fibrosis (n=6 each group), 25% CCl4 was dissolved using olive oil, and then, the mice were intraperitoneally injected with CCl4 (0.5 ml/kg body weight, twice per week for 4 or 8 weeks) or an equivalent amount of olive oil. Two days after the last injection, the rats and mice were sacrificed, and livers and serum were obtained for further study.

**Adenovirus-mediated KLF14 overexpression in rats and GW9662 administration**

For KLF14 overexpression *in vivo*, the KLF14-expressing adenovirus (Ad-KLF14) and the relevant control adenovirus vector (Ad-Vector) were purchased from Genechem (Shanghai, China). We randomly divided the rats into 4 groups (n=8 each group), and designated as group I (control), group Ⅱ (Ad-Vector), group Ⅲ (Ad-KLF14) and group Ⅳ (Ad-KLF14+ GW9662), respectively. For group Ⅱ, Ⅲ and Ⅳ, rats received intraperitoneal injection of TAA (150 mg/kg body weight) twice per week for 8 weeks, while rats of group I were injected with an equivalent volume of saline. After 4 weeks, each rat in group Ⅲ and Ⅳ was injected with Ad-KLF14 via the tail vein (5×10^9^ plaque forming units diluted in 200 μl saline) three times at an interval of 10 days in the last 4 weeks, while rats of groupI and Ⅱ received an equivalent volume of saline or Ad-Vector, respectively. In addition, rats of group Ⅳ were intraperitoneally injected with GW9662 (3 mg/kg body weight, dissolved using DMSO and diluted in saline^[3](#_ENREF_3)^) daily for 8 weeks.

**EPZ-6438 administration *in vivo***

We randomly divided the rats into 3 groups (n=8 each group), and designated as the group I (control), group Ⅱ (TAA), and group Ⅲ (TAA+EPZ-6438), respectively. The rats in group Ⅱ, Ⅲ were intraperitoneally injected with TAA (150 mg/kg body weight) twice per week for 8 weeks, while rats in group I received equivalent amount of saline. In group Ⅲ, each rat received oral delivery of EPZ-6438 (350 mg/kg body weight, dissolved in 0.5% NaCMC containing 0.1% Tween-80 in water)^[4](#_ENREF_4),[5](#_ENREF_5)^ or vehicle 5 days per week during the last 6 weeks.

**Immunohistochemistry (IHC) and immunofluorescence (IF) staining**

The detailed procedures of IHC and IF staining were described previously.^[2](#_ENREF_2)^ For IHC staining, the human, rat and mouse liver tissues were stained with antibodies against α-SMA or KLF14. For double IF staining, the human liver tissues were stained with antibodies against α-SMA or KLF14. The paraffin-embedded liver sections from the *in vivo* assay were incubated with FITC-Ki67 antibody and Cy3-α-SMA antibody to detect the proliferative HSCs *in vivo.* DAPI (Promoter, Wuhan, China) was used to stain the nuclei. Digital images were obtained using a fluorescence microscope (Olympus, Japan). Detailed antibody information is presented in Table S2.

**Oil Red O staining**

The indicated LX-2 and HSC-T6 cells were seeded on sterile coverslips in 24-well plates and treated accordingly. The lipid droplets (LD) content was assessed by Oil Red O staining following the standard procedures. Briefly, after fixation by 4% paraformaldehyde (Promoter, wuhan, China), permeabilization by 0.3% Triton X-100 (Promoter), and differentiation by 60% isopropanol, the cells were stained with pre-warmed and filtered Oil Red O solution (Servicebio technology, wuhan, China) for 30 min. Then, the cells were incubated in 60% isopropanol for differentiation, and washed with distilled water. Then, the cells were incubated with hematoxylin (Servicebio) for 15 sec and washed with distilled water. Coverslips were then mounted with aqueous mounting media, and images were acquired using a microscope (Olympus). Each group included at least three replicates.

**HSCs proliferation measurement**

The indicated HSCs were seeded in 96-well plates and treated accordingly. HSCs proliferation was assessed by Cell Counting Kit-8 analysis (CCK-8) (Promoter), as previously described.^[2](#_ENREF_2)^ In addition, HSCs proliferation was assessed by 5-ethynyl-2′-deoxyuridine (EdU) incorporation assay using EdU kits (Riobio, Guangzhou, China) following the standard protocol. Briefly, indicated LX-2 cells were seeded in 96-well plates with a density of 4×10^3^ cells each well, and treated accordingly. Then, the cells were incubated with the EdU buffer (50 μM) at 37°C for 2 h. After fixation by 4% paraformaldehyde for 0.5 h and permeabilization by 0.3% Triton X-100 for 10 min, the cells were stained with EdU solution, subsequently, the Hoechst 33342 was added into culture to stain the nuclei. Then, images were acquired using a fluorescence microscope (Olympus). Each group included at least three replicates.

**Flow cytometry analyses of cell cycle and apoptosis**

The flow cytometry analyses of cell cycle and apoptosis were performed as previously described.^[2](#_ENREF_2)^ For cell cycle analysis, the indicated cells were collected and fixed with 75% ethanol at 4 °C overnight, then treated with RNase A (Promoter) and stained with propidium iodide (Promoter) for 30 min at 37 °C. Cell cycle profiles were determined using BD FACS Verse (BD BioScience, CA, USA). For the analysis of cell apoptosis, the indicated cells were harvested and stained with PE-Annexin-V/7-AAD Cell Apoptosis Kit (BD BioScience) for 15 min in darkness at room temperature. The apoptotic rates of the cells were then quantified using BD FACS Verse (BD BioScience). The experiments were performed independently at least three times.

**Transwell migration assay**

HSCs migratory ability was assessed by Transwell migration assay, as previously described.^[6](#_ENREF_6)^ Briefly, 2×10^4^ indicated cells were resuspended in 200 μl DMEM (serum-free), and then plated in the upper chamber (8-μm pore size, Corning, NY, USA), 600 μl DMEM containing 20% FBS was added to the bottom chamber. After 48 h, cells which had migrated to the lower layers were fixed with methanol and stained using crystal violet. The number of migratory cells was counted under an invert microscope (Olympus). Each group included three replicates.

**Real-time quantitative PCR (****RT-qPCR)**

The total RNA extraction and RT-qPCR were performed, as previously described.^[2](#_ENREF_2)^ The primer sequences are presented in Table S1.

**Western blotting**

The total protein extraction and Western blotting analysis were performed, as previously described.^[2](#_ENREF_2)^ Detailed antibody information is presented in Table S2.

**Plasmid construction**

Standard procedures for plasmid construction were described previously.^[7](#_ENREF_7)^ Relevant primers are listed in Table S1. For instance, the (-811/+200) PPARγ promoter construct was generated using human genomic DNA. This construct corresponds to the sequence from -811 to +200 (relative to the transcriptional start site) of the 5’-flanking region of the human PPARγ gene. It was generated with forward and reverse primers incorporating KpnI and XhoI sites at the 5’ and 3’-ends, respectively. The PCR product was cloned into the KpnI and XhoI sites of the pGL3-Basic vector (Promega, USA). The 5’-flanking deletion constructs of the PPARγ promoter, (-460/+200) PPARγ, (-227/+200) PPARγ, (-98/+200) PPARγ, were similarly generated using the (-811/+200) PPARγ construct as the template. The KLF14 binding sites in the PPARγ promoter were mutated using the QuikChange II Site-Directed Mutagenesis Kit (Stratagene, CA, USA). The constructs were confirmed by DNA sequencing.

**Construction of lentivirus and stable cell lines**

Lentiviral vectors encoding shRNAs were generated using PLKO.1-TRC (Addgene) and designated as LV-shPPARγ, LV-shEZH2, LV-shKLF14 and LV-shcontrol. “LV-shcontrol” is a non-target shRNA control. The vector “PLKO.1-puro Non-Target shRNA Control Plasmid DNA” (purchased from Sigma, SHC016) contains an shRNA insert that does not target any known genes from any species. The shRNA sequences can be found in Table S1. Lentiviral vectors encoding the human and rat KLF14 genes were constructed in FUW-teto (Addgene) and designated as LV-KLF14. An empty vector was used as the negative control and was designated as LV-control. The multiplicity of infection (MOI) for lentivirus transfection is 100, in the presence of polybrene. Seventy-two hours after infection, puromycin (OriGene) was applied for HSCs cells selection for 2 weeks. Selected pools of knockdown and overexpressing cells were used for the following experiments.

**Transient transfection**

The serum-starved LX-2 cells were plated at a density of 1×10^5^cells/well in a 24-well plate. After 12-24 hours, the cells were co-transfected with 0.6 μg of the expression vector plasmids, 0.18 μg of the promoter reporter plasmids, and 0.02 μg of the pRL-TK plasmids using Lipofectamine 3000 (Invitrogen, USA) according to the manufacturer’s instructions. After 5 h of transfection, the cells were washed and allowed to recover overnight in fresh medium supplemented with 1% FBS for 48 h.

**Luciferase reporter assay**

Luciferase activity was detected using the Dual Luciferase Assay (Promega, USA) according to the manufacturer’s instructions. The transfected cells were lysed in culture dishes containing a lysis buffer, and the resulting lysates were centrifuged at maximum speed for 1 min in an Eppendorf microcentrifuge. Relative luciferase activity was determined using a Modulus^TM^ TD20/20 Luminometer (Turner Biosystems, USA), and the transfection efficiency was normalized according to the Renilla activity. The experiments were independently performed at least three times.

**Chromatin immunoprecipitation analysis (ChIP)**

The ChIP analysis was performed using an EZ-Magna ChIP A/G Kit (Millipore), as previously described.^[7](#_ENREF_7)^ Briefly, indicated LX-2 and R-HSCs cells were crosslinked with 1% formaldehyde for 10 minutes at 37℃. After cell lysis, the DNA was fragmented by sonication, the immunoprecipitation was performed using antibodies against KLF14, EZH2, H3K27me3 and isotype control IgG. Then, qRT-PCR was performed to amplify the corresponding binding site within the promoters. The primers used for amplification are shown in Table S1, and detailed antibody information is presented in Table S2. The experiments were independently performed at least three times.

.

**Hydroxyproline assay**

Total collagen content was evaluated by measuring the amount of hydroxyproline in rat liver tissues (n=8) using a commercially available hydroxyproline detection kit (Jiancheng, Nanjing, China) according to the manufacturer’s instructions.

**Liver enzyme measurement**

The alanine aminotransferase (ALT) and aspartate aminotransferase (AST) levels in the rat serum (n=8) were determined using commercial assay kits (Jiancheng, Nanjing, China) according to the manufacturer’s instructions.

**Reference**

1. Bedossa P, Poynard T. An algorithm for the grading of activity in chronic hepatitis C. The METAVIR Cooperative Study Group. *Hepatology*. 1996; 24(2):289-293.

2. Fan Y, Du Z, Steib CJ, et al. Effect of SEPT6 on the biological behavior of hepatic stellate cells and liver fibrosis in rats and its mechanism. *Lab Invest*. 2019; 99(1):17-36.

3. Wang CY, Liu Q, Huang QX, et al. Activation of PPARgamma is required for hydroxysafflor yellow A of Carthamus tinctorius to attenuate hepatic fibrosis induced by oxidative stress. *Phytomedicine*. 2013; 20(7):592-599.

4. Zhang H, Zhu D, Zhang Z, et al. EZH2 targeting reduces medulloblastoma growth through epigenetic reactivation of the BAI1/p53 tumor suppressor pathway. *Oncogene*. 2020; 39(5):1041-1048.

5. Jin, X, Kim, LJY, Wu, Q, et al. Targeting glioma stem cells through combined BMI1 and EZH2 inhibition. *Nat Med*. 2017; 23(11):1352-1361.

6. Du Z, Lin Z, Wang Z, Liu D, Tian D, Xia L. SPOCK1 overexpression induced by platelet-derived growth factor-BB promotes hepatic stellate cell activation and liver fibrosis through the integrin alpha5beta1/PI3K/Akt signaling pathway. *Lab Invest*. 2020; 100(8):1042-1056.

7. Xia L, Huang W, Tian D, et al. Upregulated FoxM1 expression induced by hepatitis B virus X protein promotes tumor metastasis and indicates poor prognosis in hepatitis B virus-related hepatocellular carcinoma. *J Hepatol*. 2012; 57(3):600-612.

**Figure S1**

**
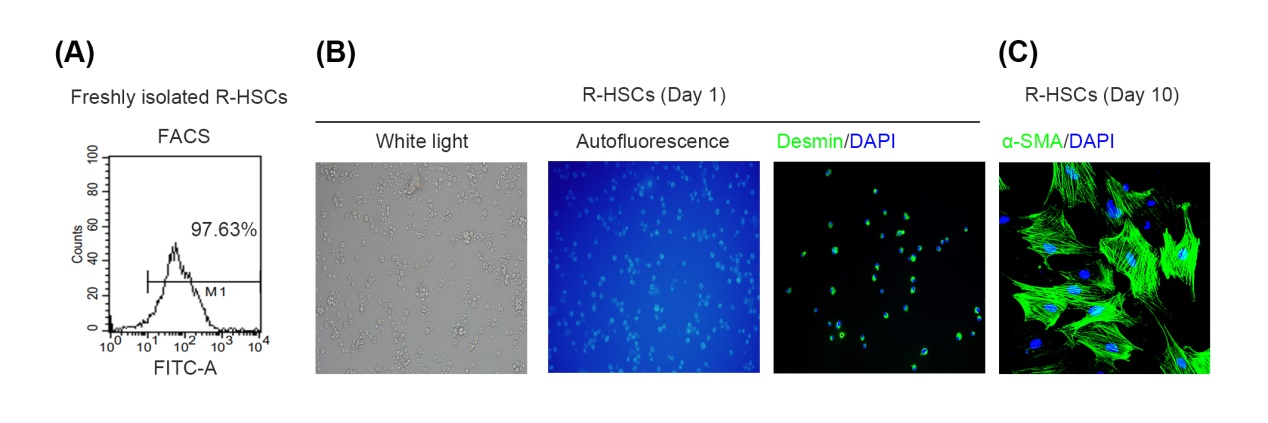
**

**Figure S1 Identification of the rat primary HSCs (R-HSCs) .**

A, The purity of freshly isolated rat primary HSCs were mearsured by flow cytometry analysis.

B, Immunofluorescence staining of Desmin in quiescent R-HSCs (1day), and quiescent R-HSCs had blue-green autofluorescence under ultraviolet excitation. (Scale bars: 50 μm)

C, Immunofluorescence staining of α-SMA in activated R-HSCs (10 day). (Scale bars: 20 μm)

**Figure S2**

**
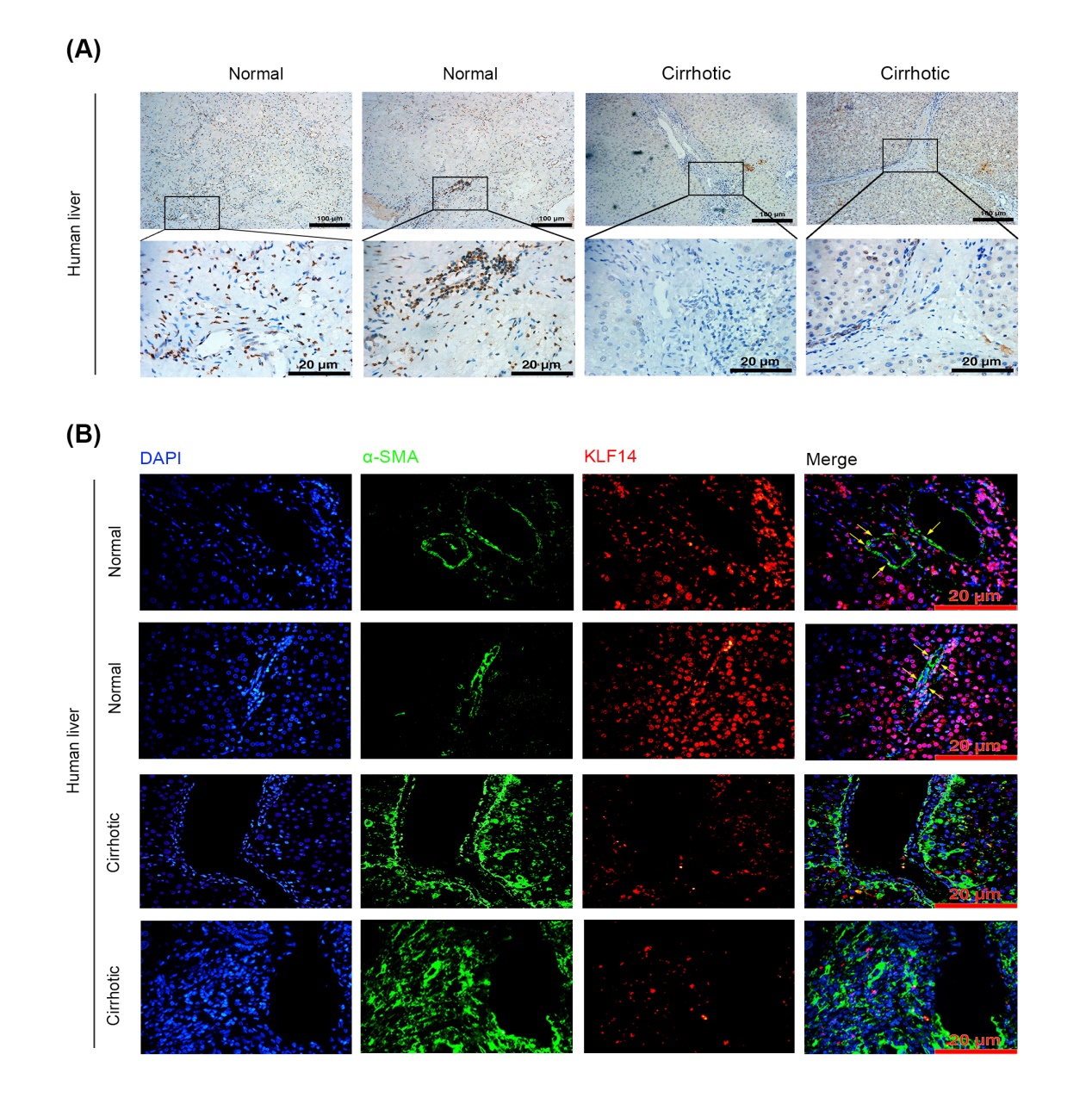
**

**Figure S2 Representative images of immunohistochemistry (IHC) and immunofluorescence (IF) staining for KLF14 and α-SMA obtained with other donors.**

A, Immunohistochemistry staining of KLF14 in human liver tissues (normal and cirrhotic, n=3, Scale bars: 100 μm and 20 μm).

B, Immunofluorescence staining of KLF14 and α-SMA in human liver tissues (normal and cirrhotic, n=3, Scale bars: 20 μm). Yellow arrows indicated HSCs.

**Figure S3**


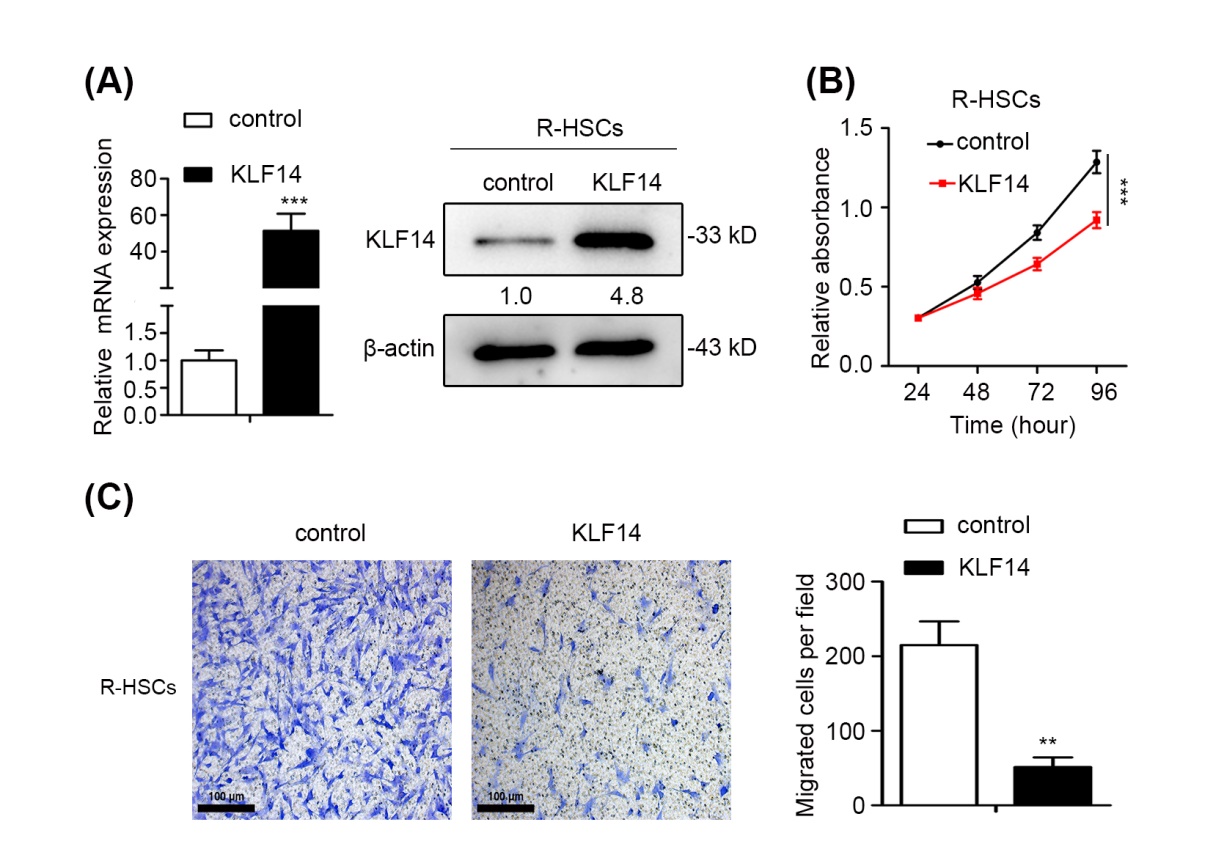


**Figure S3 KLF14 overexpression inhibits proliferation and migration of activated R-HSCs.**

A, The activated R-HSCs were transfected with rat KLF14-expressing lentivirus (LV-KLF14) or relevant control lentivirus (LV-control), 72 h later, mRNA and protein were extracted, and RT-qPCR and Western blotting analyses were performed to measure KLF14 epxression.

B, Cell proliferation was assessed by CCK-8 assay.

C, Cell migration ability was measured by Transwell migration assay (Scale bars: 100 μm).

***P*< 0.01, ****P*< 0.001 vesus the control group. n=3

**Figure S4**


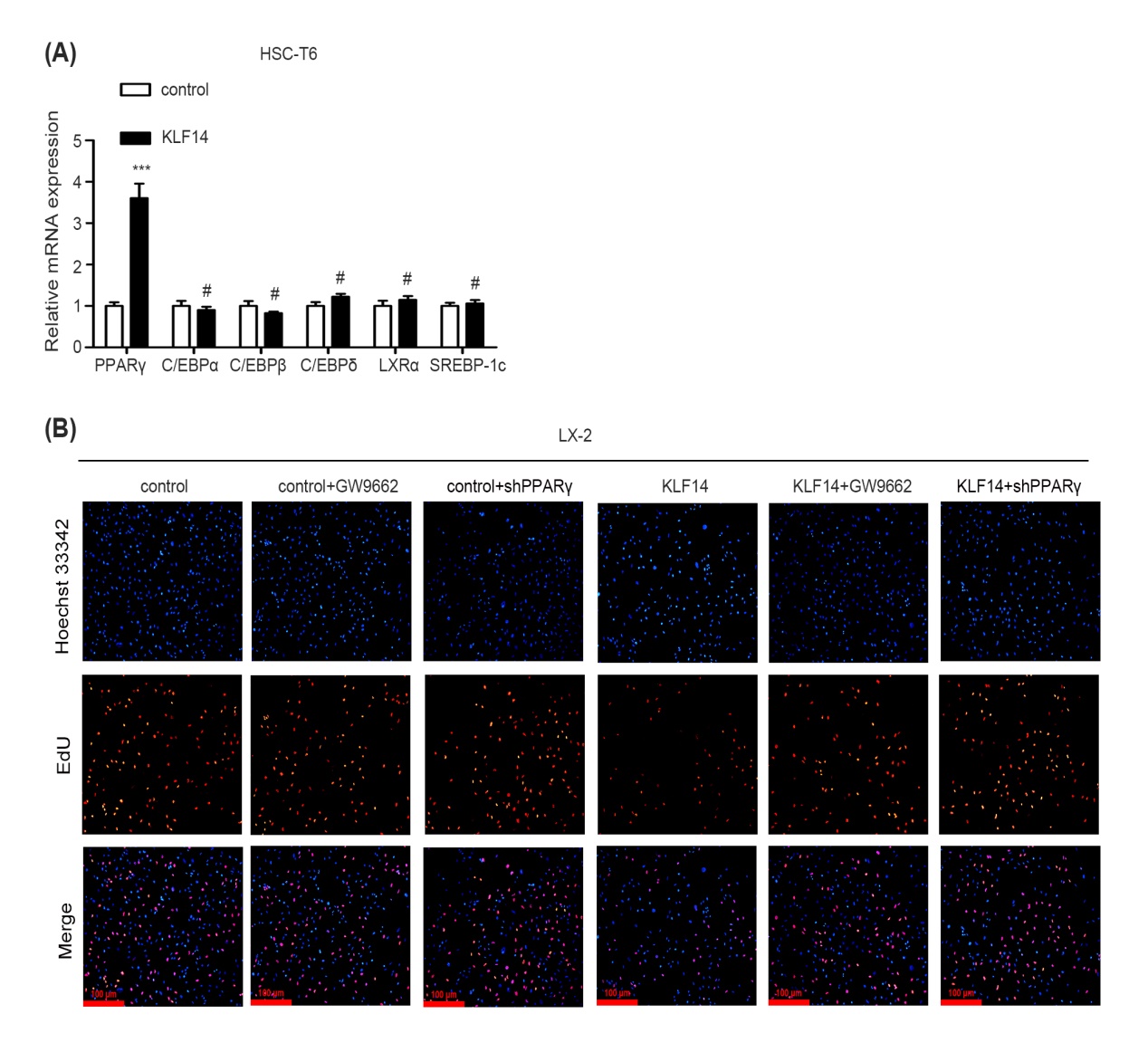


**Figure S4 PPARγ is essential for the inhibitory role of KLF14 overexpression in HSCs.**

A, The mRNA levels of PPARγ, C/EBPα, C/EBPβ, C/EBPδ, LXRα and SREBP-1c in indicated HSC-T6 cells were measured by RT-qPCR analysis.

B, EdU incorporation assay of the indicated LX-2 cells was performed to measured cell proliferation (Scale bars: 100 μm).

^***^*P*< 0.001, ^#^*P*＞0.05 vesus the control group. n=3

**Figure S5**


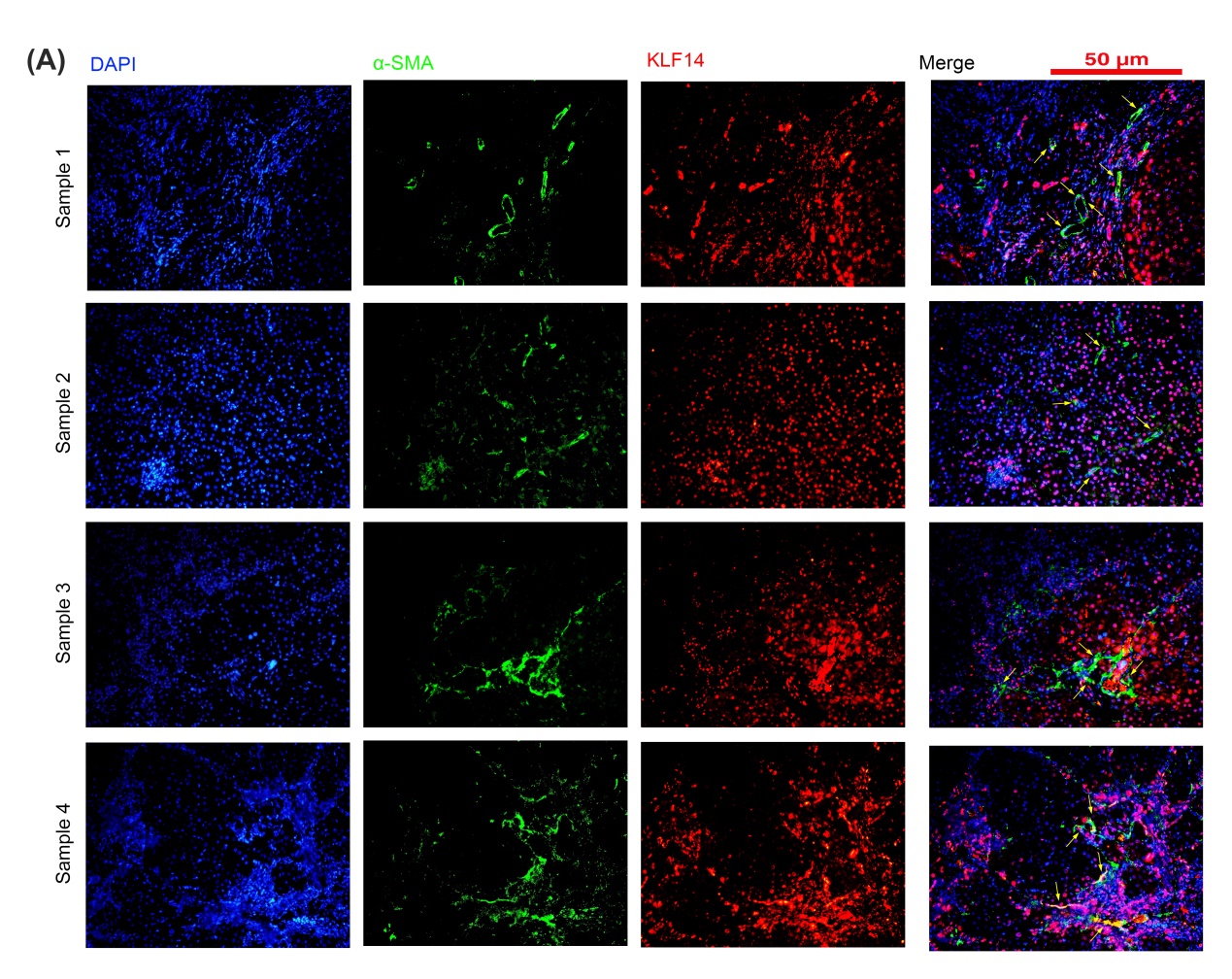


**Figure S5 Injection of KLF14-expressing adenovirus targets both hepatocytes and HSCs.**

A, Representative images of co-immunoflurescence staining of KLF14 and α-SMA in KLF14-expressing adenovirus treated rats. Yellow arrows indicated HSCs. (n=4, Scale bars: 50 μm).

**Figure S6**


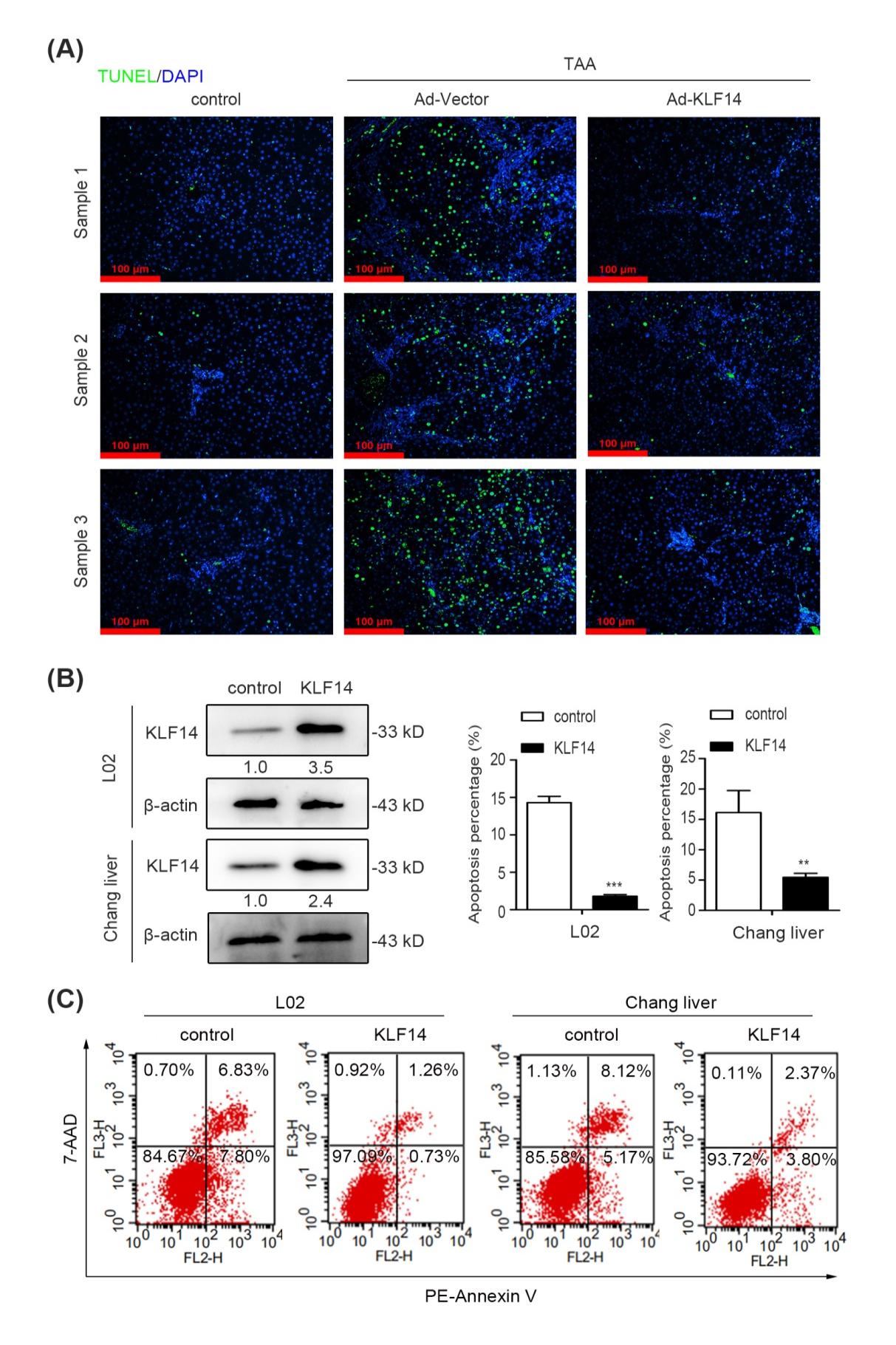


**Figure S6 KLF14 overexpression inhibits apoptosis of hepatocytes *in vivo* and *in vitro*.**

A, FITC-conjugated TUNEL (green) assay was performed to measure the apoptotic cells in rats. Nuclei were couterstained by DAPI (n=3, Scale bars: 100μm).

B, KLF14 expression in the indicated hepatocytes was measured by Western blotting analysis.

C, Cell apoptosis was analyzed by flow cytometry.

^**^*P*< 0.01, ^***^*P*< 0.001 vesus the control group. n=3

**Figure S7**


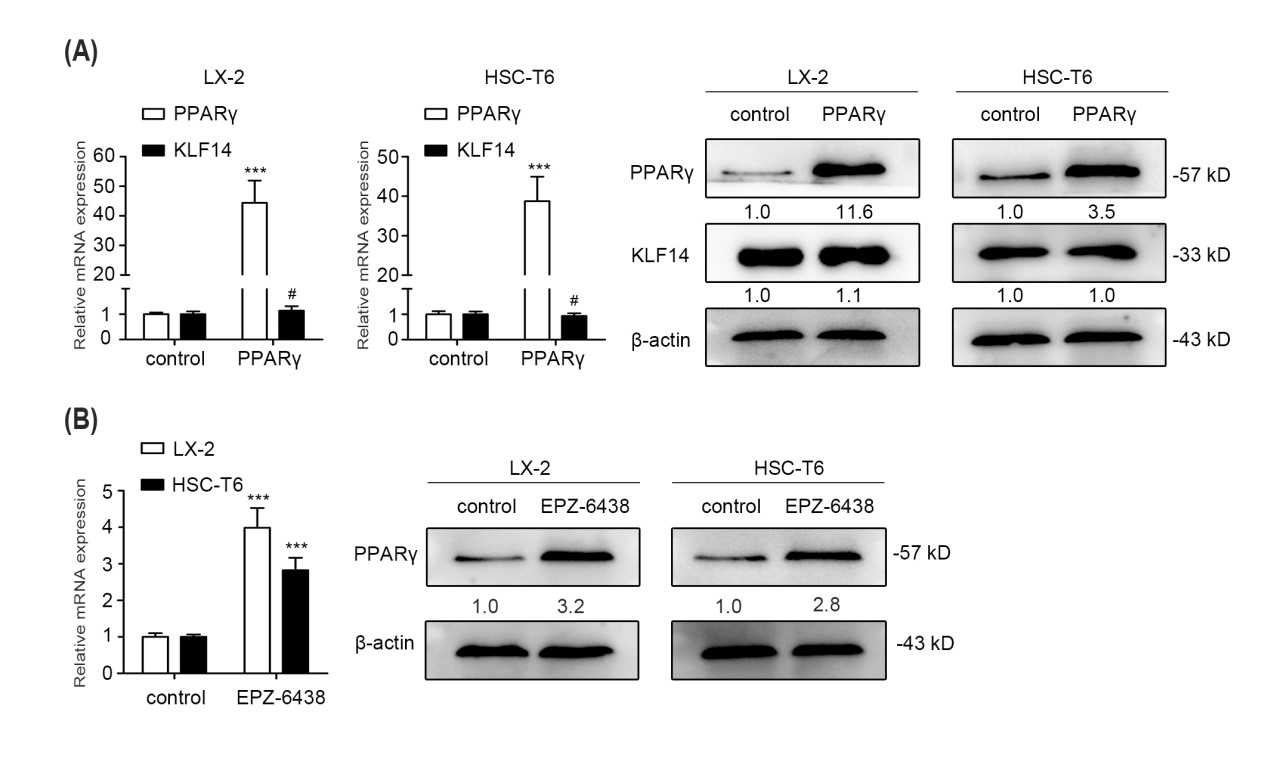


**Figure S7 PPARγ doesn’t regulate KLF14 expression, and EZH2 inhibitor EPZ-6438 induced PPARγ expression.**

A, LX-2 and HSC-T6 cells were infected with human and rat PPARγ-expressing lentivirus, 72 h later, the expression of PPARγ and KLF14 were confirmed by RT-qPCR and Western blotting analyses.

B, LX-2 and HSC-T6 cells were treated with EZH2 inhibitor (EPZ-6438, 10 μM) for 72h, and then, PPARγ expression was measured by RT-qPCR and Western blotting analyses.

****P*< 0.001, *#P*＞0.05 vesus the control group. n=3

**Figure S8**


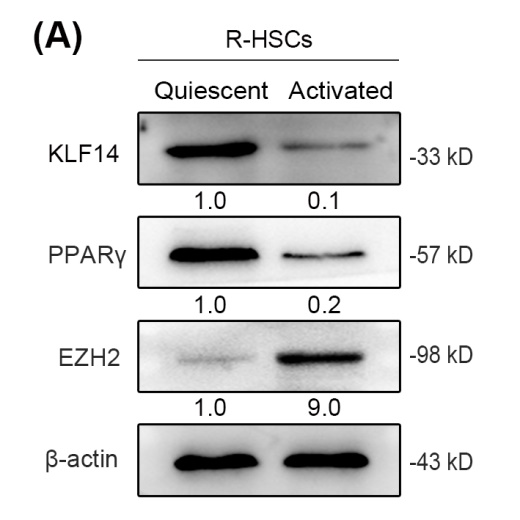


**Figure S8 Inverse expression of KLF14/PPARγ and EZH2 in quiescent and activated R-HSCs.**

A, Protein levels of KLF14, PPARγ and EZH2 were measured by Western blotting analysis. ( n=3)

**Table S1. Primer and shRNA sequences used in the study**

| **Primer name** | **Primer sequences** | **Enzyme** |
| --- | --- | --- |
| **Primers for real-time PCR（human）:** |  |  |
| KLF14 sense: | 5'-GGAACTGATAAGAAGGGATGAA-3' |  |
| KLF14 antisense: | 5'- CCTGGTGGATGGGTGAGACA-3' |  |
| α-SMA sense: | 5’-GTGTTGCCCCTGAAGAGCAT -3’ |  |
| α-SMA antisense: | 5’-GCTGGGACATTGAAAGTCTCA-3’ |  |
| COL1A1 sense: | 5’-ATCCGAGGAGCTACTAGAGGG-3’ |  |
| COL1A1 antisense: | 5’-AGCGCAGATGCTCGTACTTG-3’ |  |
| PPARγ sense | 5'-GCTGGCCTCCTTGATGAAT -3' |  |
| PPARγ antisense | 5'-CACATTCAGCAAACCTGGG -3' |  |
| C/EBPα sense | 5'-TTGTATCTGGCCTCTGTGCC-3' |  |
| C/EBPα antisense | 5'-GCCGACGGAGAGTCTCATTT-3' |  |
| C/EBPβ sense | 5'- CGACGAGTACAAGATCCGGC-3' |  |
| C/EBPβ antisense | 5'-TGCTTGAACAAGTTCCGCAG-3' |  |
| C/EBPδ sense | 5'-ATGAGCGCCGCGCTCTTCAGCCTGGA-3' |  |
| C/EBPδ antisense | 5'-CGTTACCGGCAGTCTGCTGTCCCG-3' |  |
| LXRα sense | 5'-GGCCCTGCATGCCTATGT-3' |  |
| LXRα antisense | 5'-CATTAGCATCCGTGGGAACA-3' |  |
| SREBP-1c sense | 5'-CGGCGCTGCTGACCGACATC-3’ |  |
| SREBP-1c antisense | 5'-CCCTGCCCCACTCCCAGCAT-3’ |  |
| EZH2 sense | 5'-TTGTTGGCGGAAGCGTGTAAAATC-3’ |  |
| EZH2 antisense | 5'-TCCCTAGTCCCGCGCAATGAGC-3 |  |
| GAPDH sense: | 5’-GCACCGTCAAGGCTGAGAAC-3’ |  |
| GAPDH antisense: | 5’-TGGTGAAGACGCCAGTGGA-3’ |  |
| **Primers for real-time PCR（Rat）:** |  |  |
| KLF14 sense: | 5'-GCGAGGATGAGCTCTCTGAC-3' |  |
| KLF14 antisense: | 5'-GCTTTGTTGCAGCCATGGAA-3' |  |
| α-SMA sense: | 5’-CCGAGATCTCACCGACTACC-3’ |  |
| α-SMA antisense: | 5’-TCCAGAGCGACATAGCACAG-3’ |  |
| COL1A1sense: | 5’-AGCTGCATACACAATGGCCTAA-3’ |  |
| COL1A1 antisense: | 5’-CCTATGACTTCTGCGTCTGGTG-3’ |  |
| PPARγ sense | 5’-TTCATGTGGCCTGTTGTAGAG-3’ |  |
| PPARγ antisense | 5’-GACCTGAAGCTCCAAGAATACC-3’ |  |
| C/EBPα sense | 5'-GGCGGGAACGCAACAA-3' |  |
| C/EBPα antisense | 5'-TCCACGTTGCGCTGTTTG-3' |  |
| C/EBPβ sense | 5'-AAGCTGAGCGACGATACAAG-3' |  |
| C/EBPβ antisense | 5'-GTCAGCTCCAGCACCTTGTG-3' |  |
| C/EBPδ sense | 5'- CGACCCCTGCCATGTATGAC-3' |  |
| C/EBPδ antisense | 5'-GGCAGCCATGGAATCAATGT-3' |  |
| LXRα sense | 5'-ATGCCTATGTCTCCATCAACCA-3' |  |
| LXRα antisense | 5'-AGAGCAGAGGGGGAAGTTTTT-3' |  |
| SREBP-1c sense | 5'-GCAACACTGGCAGAGATCTACGT-3 |  |
| SREBP-1c antisense | 5'-TGGCGGGCACTACTTAGGAA -3 |  |
| GAPDH sense: | 5’-AACGGCACAGTCAAGGCTGA-3’ |  |
| GAPDH antisense: | 5’-ACGCCAGTAGACTCCACGACAT-3’ |  |
| **Primers for real-time PCR（Mouse）:** |  |  |
| KLF14 sense: | 5'-CTCCGTGTGCCTCAACTAGC-3' |  |
| KLF14 antisense: | 5'-CAGGCGCATCCAGGATAGC-3' |  |
| α-SMA sense: | 5’-CCCAGACATCAGGGAGTAATGG-3’ |  |
| α-SMA antisense: | 5'-TCTATCGGATACTTCAGCGTCA-3' |  |
| GAPDH sense: | 5'-AGGTCGGTGTGAACGGATTTG -3' |  |
| GAPDH antisense: | 5’-TGTAGACCATGTAGTTGAGGTCA-3’ |  |
|  |  |  |
| Primers for PPARγ promoter construct: | |  |
| (-811/+200) PPARγ sense: | 5’-TATAGGTACCTGATTGCGCCACAGCACT-3’ | KpnI |
| (-460/+200) PPARγ sense: | 5’-TATAGGTACCAACGAGGACCTTCTGTAC-3’ | KpnI |
| (-227/+200) PPARγ sense: | 5’-TATAGGTACCCACTTTGGACAGGTCACG-3’ | KpnI |
| (-98/+200) PPARγ sense: | 5’-TATAGGTACCGGTGTCAGAAACACTGCT-3’ | KpnI |
| antisense: | 5’-ATATCTCGAGGTGTATCAGCAGTTCCAC-3’ | XhoI |
| Primers for PPARγ promoter site-directed mutagenesis:  KLF14 binding site: | |  |
| binding site 3 mutation sense: | 5’-GACACAGCAACAaaaTGTCTCATGG-3’ |  |
| binding site 3 mutation antisense: | 5’-CCATGAGACAtttTGTTGCTGTGTC-3’ |  |
| binding site 2 mutation sense: | 5’-AAATGCAGAGCAaaaCAGCATACAG-3’ |  |
| binding site 2 mutation antisense: | 5’-CTGTATGCTGtttTGCTCTGCATTT-3’ | |
| binding site 1 mutation sense: | 5’-ACTCCGTCCCCAaaaATGTTGTCTG-3’ | |
| binding site 1 mutation antisense: | 5’-CAGACAACATtttTGGGGACGGAGT-3’ |  |
| Primers used for ChIP in the PPARγ promoter: | | |
| distant region sense: | 5’-ACACCGAGTTACCTTATG-3’ |  |
| distant region antisense: | 5’-ACAAGGTTACTTGTGGAC-3’ |  |
| binding site 3 sense: | 5’-TGGGAGGATTCCTTGAGC-3’ |  |
| binding site 3 antisense: | 5’-TCCAGAGTTCCAATCTGC-3’ |  |
| binding site 2/1 sense: | 5’-GAGGACCTTCTGTACCAG-3’ |  |
| binding site 2/1 antisense: | 5’-CAGTGTTTCTGACACCGA-3’ |  |
| Primers used for ChIP in the KLF14 promoter: | |  |
| Primer 1 sense: | 5’-AACTTTCTGGGACTCCGC-3’ |  |
| Primer 1 antisense: | 5’-CCGGCTAAGTCATGTTTA-3’ |  |
| Primer 2 sense: | 5’-TCTGGTTCGAGATGCTAT-3’ |  |
| Primer 2 antisense: | 5’-GCGATTGTTAGCGAAGTG-3’ |  |

**shRNA sequences**

| Gene name | shRNA sequences |
| --- | --- |
| PPARγ | 5'-CCGGCAGCATTTCTACTCCACATTACTCGAGTAATGTGGAGTAGAAATGCTGTTTTT -3' |
| EZH2 | 5'-CCGGCCCAACATAGATGGACCAAATCTCGAGATTTGGTCCATCTATGTTGGGTTTTTG -3' |
| KLF14 | 5'-CCGGGCGAGCGCTTTGGCTGATTTACTCGAGTAAATCAGCCAAAGCGCTCGCTTTTT-3' |

**Table S2. Antibodies used in the study**

Primary Antibodies for WB

| **Antibody** | **Concentration** | **Catalog** | **Company** |
| --- | --- | --- | --- |
| KLF14 | 1:400 | SAB1304202 | Sigma-Aldrich |
| α-SMA | 1:10000 | ab124964 | Abcam |
| COL1A1 | 1:300 | bs10423R | Bioss |
| PPARγ | 1:1000 | ab209350 | Abcam |
| EZH2 | 1:1000 | 5246 | Cell Signaling Technology |
| β-actin | 1:10000 | 66009-1-Ig | Proteintech |
| H3K27me3 | 1:1000 | 9733 | Cell Signaling Technology |
| Histone H3 | 1:2000 | 4499 | Cell Signaling Technology |
| Anti-Rabbit IgG | 1:3000 | 111-035-003 | Jackson |
| Anti-mouse IgG | 1:3000 | 115-035-003 | Jackson |

**Primary Antibodies for IHC & IF**

| Antibody | Concentration | Catalog | Company |
| --- | --- | --- | --- |
| α-SMA | 1:1000 (IHC) | ab124964 | Abcam |
| KLF14 | 1:100 (IHC &IF) | SAB1304202 | Sigma-Aldrich |
| α-SMA | 1:100 (IF) | BM0002 | Boster |
| FITC-Ki67 | 1: 100 (IF) | 612472 | BD Bioscience |
| Cy3-α-SMA | 1:100 (IF) | C6198 | Sigma-Aldrich |
| Desmin | 1:50 (IF) | GM063002 | Genetek |

**Primary Antibodies for ChIP**

| Antibody | Concentration | Catalog | Company |
| --- | --- | --- | --- |
| KLF14 | 2 μg/reaction tube | PCRP-KLF14-1H1 | Developmental Studies Hybridoma Bank |
| EZH2 | 1:100 | 5246 | CellSignaling Technology |
| H3K27me3 | 1:50 | 9733 | Cell Signaling Technology |
| Isotype IgG (anti-rabbit) | 2.5 mg/ml | 3900 | Cell Signaling Technology |
| Isotype IgG (anti-mouse) | 2.5 mg/ml | 37988 | Cell Signaling Technology |
